# A Study of the Resistance of Hu Sheep Lambs to *Escherichia coli* F17 Based on Whole Genome Sequencing

**DOI:** 10.3390/ani14010161

**Published:** 2024-01-03

**Authors:** Yanjun Duan, Pengwei Su, Yifei Gu, Xiaoyang Lv, Xiukai Cao, Shanhe Wang, Zehu Yuan, Wei Sun

**Affiliations:** 1 College of Veterinary Medicine, Yangzhou University, Yangzhou 225009, China; yjduan1996@163.com; 2 College of Animal Science and Technology, Yangzhou University, Yangzhou 225009, China; supengwei4901@163.com (P.S.); feilin133082@163.com (Y.G.); 007121@yzu.edu.cn (S.W.); 3 Joint International Research Laboratory of Agriculture and Agri-Product Safety of Ministry of Education of China, Yangzhou University, Yangzhou 225009, China; dx120170085@yzu.edu.cn (X.L.); cxkai0909@163.com (X.C.); yuanzehu1988@163.com (Z.Y.); 4 International Joint Research Laboratory in Universities of Jiangsu Province of China for Domestic Animal Germplasm Resources and Genetic Improvement, Yangzhou University, Yangzhou 225009, China

**Keywords:** whole genome sequencing, *Escherichia coli* F17, Hu sheep lambs, antagonistic phenotype, susceptible phenotype

## Abstract

**Simple Summary:**

Acute infectious diseases caused by *Escherichia coli* in lambs or young sheep have severe impacts on the growth and development of sheep, among which *Escherichia coli* F17 (*Escherichia coli* F17, *E. coli* F17) is one of the primary pathogens causing bacterial diarrhoea in lambs. The mechanism of lambs’ resistance regulation to it remains to be elucidated.

**Abstract:**

This study aims to analyze the whole genome sequencing of *E. coli* F17 in antagonistic and susceptible Hu sheep lambs. The objective is to investigate the critical mutation loci in sheep and understand the genetic mechanism of sheep resistance to *E. coli* F17 at the genome level. Antagonist and susceptible venous blood samples were collected from Hu sheep lambs for whole genome sequencing and whole genome association analysis. A total of 466 genes with significant SNPs (*p* < 1.0 × 10^−3^) were found. GO and KEGG enrichment analysis and protein interaction network analysis were performed on these genes, and preliminary investigations showed that SNPs on CTNNB1, CDH8, APOD, HCLS1, Tet2, MTSS1 and YAP1 genes may be associated with the antagonism and susceptibility of Hu sheep lambs to *E. coli* F17. There are still some shortcomings that have not been explored via in vivo and in vitro functional experiments of the candidate genes, which will be our next research work. This study provides genetic loci and candidate genes for resistance of Hu sheep lambs to *E. coli* F17 infection, and provides a genetic basis for breeding disease-resistant sheep.

## 1. Introduction

Sheep, one of the six domesticated animal species, occupy an important position in the livestock industry, and sheep milk, mutton, skin and fleece provide significant economic benefits to the farming industry. *E. coli* disease has a major impact on the entire sheep production cycle, particularly during the lambing and fattening period; acute infectious diseases caused by *E. coli* in lambs or young sheep seriously affect the growth and development of sheep [1].

Intestinal pathogenic *E. coli* types are enteropathogenic *E. coli* (EPEC); Shiga toxin-producing *E. coli* (STEC); enterotoxin-producing *E. coli* (ETEC); enteroaggregative *E. coli* (EAEC); enteroinvasive *E. coli* (EIEC); and diffusely adherent *E. coli* (DAEC) [2]. *E. coli* F17 is mainly detected in diarrhoeal or septic calves or lambs. F17-mediated *E. coli* is mainly composed of the structural subunit F17-A and the adhesin subunit F17-G [3]. Four structural subunits of F17-A have been identified so far: F17a-A, F17b-A, F17c-A, and F17d-A [4,5,6,7]. F17-G has been identified as three adhesin subunits: F17a-G, F17b-G and F17c-G [8]. In a related study, 45 nonenterotoxin-producing *Escherichia coli* strains from sheep and goats that produced F17-associated hyphae were characterized by hyphal structural subunits and adhesin subtypes; the majority of the sheep and goat strains showed phenotypic characteristics of the septic strain [9]. In addition, *E. coli* F17 was detected in the diarrheal feces of lambs in several regions, indicating that *E. coli* F17 is a pathogenic pathogen for lambs [10].

ETEC adapts to its environment in various ways, including by the development of different strains that express multiple colonization factors (CFs) that adhere to the intestinal epithelium and secrete numerous enterotoxins. To avoid ETEC infection, it is necessary to have a deep understanding of its pathogenic mechanism. ETEC secretes colonization factors (CFs) and enterotoxin, two virulence factors which bind to specific receptors of intestinal epithelial cells to induce diarrhoea [11]. In addition, intestinal epithelial cells (IECs) recognize pathogen-associated molecular patterns (PAMPs) through pathogen recognition receptors (PRRs), which secrete a variety of cytokines and chemokines to trigger innate immune defences and promote adaptive immune responses [12,13]. In ETEC infection, the integrity of the intestinal barrier plays a vital role in resisting the attack of various pathogenic microorganisms [14,15]. The physical barrier formed by intestinal epithelial cells and tight intercellular connections is the first line of defence against harmful substances and the basis for maintaining the function and structure of the intestinal barrier [16]. Mucin secreted by goblet cells in intestinal epithelial cells forms a mucus layer, separating intestinal lumen microorganisms from epithelial cells and preventing the invasion of toxins and pathogenic microorganisms [17]. Therefore, the colonization and adhesion of ETC in intestinal epithelial cells, binding to specific receptors, maintenance of the intestinal barrier function, and activation of the immune system can affect the invasion of ETEC into the body.

To fundamentally solve bacterial diarrhoea caused by *E. coli* F17 in lambs, we should understand the mechanism of diarrhoea and implement disease-resistance breeding accordingly. In this study, we performed whole genome sequencing of antagonistic and susceptible sheep individuals at the genomic level to screen the key SNPs’ resistant to *E. coli* F17 infection and provide theoretical support for breeding resistance to the disease.

## 2. Materials and Methods

### 2.1. Ethical Statement

All animal experiments were reviewed and approved by the Experimental Animal Welfare and Ethical Institute of Animal Science, Yangzhou University (No: NFNC2020-NFY-6), and were performed in accordance with the Regulations for the Administration of Affairs Concerning Experimental Animals approved by the State Council of the People’s Republic of China.

### 2.2. Sample Collection

Fifty healthy newborn lambs were randomly selected and fed lamb milk without antimicrobial additives and probiotics between 1 and 3 days of age. After 3 days, the lambs were divided into a high-dose group and a low-dose group, and the lambs in each group were challenged by gavage with 50 mL and 1 mL of *E. coli* F17 culture (1 × 10^9^ CFU/mL), respectively, for 4 days. Ten healthy lambs in the high-dose challenge group (antagonistic type) and ten low-dose-challenged lambs with diarrhoea (sensitive type) were selected, according to intestinal pathological sections and the amount of bacterial adherence in the intestinal contents. Six lambs were selected as case and control groups in the antagonistic and sensitive groups [18]. Two mL of venous blood was taken in EDTA-K2 anticoagulant tubes and stored at −80 °C. Samples were named as containing A for the antagonistic type and not containing A for the susceptible type.

### 2.3. Sheep Blood Genomic DNA Extraction and Sequencing

Spectrophotometer, Qubit, and agarose gel electrophoresis were used to identify the concentration and purity of the extracted DNA, and the qualified DNA samples were randomly interrupted using Covaris to form 350–500 bp fragments after quality control. Library construction was then carried out using the TruSeq DNA LT Sample Prep kit, in which the DNA fragments were subjected to end repair, poly(A) tailing, sequencing junction addition, purification, PCR amplification, and other steps to complete the library construction. After constructing the library, a sequencer subjected the DNA fragments to bipartite sequencing. The library construction process included the following: fragment DNA, repair ends and select library size, adenylate 3′ ends, ligate adapters, enrich DNA fragments, normalize, and pool libraries. OE Biotech Co., Ltd. (Shanghai, China) performed the sequencing and analysis. The libraries were constructed with TruSeq Nano DNA LT Sample Preparation Kit (Illumina, San Diego, CA, USA).

### 2.4. Sequencing Data Filtering

After the raw data waas obtained, the sequencing data quality evaluation was conducted. The sequencing error rate, data volume, comparison rate, coverage, etc., were counted to evaluate whether the sequencing of the database construction met the standard, and follow-up analysis was carried out if the standard was met. In addition, variant information analysis was conducted, and high-quality sequences were compared to the reference genome. The variant information in the sample was detected, and the detected mutation information was analyzed and interpreted. High-throughput sequencing data off the sequencing machine had several sequencing errors, which were caused by the combined action of multiple factors such as the sequencer itself, sequencing reagents, and samples. In general, the error rate of the first few bases and ends of a sequencing fragment can be high, caused by the instability of the sequencing starting instrument and the consumption of chemical reagents during sequencing. To eliminate the effect of sequencing errors on the results, the raw data needed to be quality controlled to obtain clean reads. The preprocessing software was fastp [19]. The quality filtration criteria were as follows: removal of adapter sequences, and reads with N (non-AGCT) bases greater than or equal to 5; sliding window with 4 bases for Windows size, and excise if the average base quality value was less than 20 (see note below for base quality value algorithm); after the above filtering, reads with lengths less than 75 bp or an average base quality value less than 15 were removed.

### 2.5. Reference Genome Matching

Clean reads were aligned to reference genomes using BWA [20]; the mapping results were converted to the BAM format by SAMtools [21]. The results were de-redundant using Picard, and analyzed using Qualimap software comparison with the reference genome Oar_rambouillet_v1.0.

### 2.6. SNP Mutation Detection

SNP detection was performed using the Haplotypecaller module of GATK4 [22] software based on the comparison of the samples with the reference genome. To reduce the error rate of SNP detection, the criterion of QD >= 2.0 was chosen for filtering, and only the mutant loci that meet this criterion were retained. QD:QD is the ratio of the quality of a variant divided by the depth of coverage. This is the quality of a variant per unit of depth; most of the false positive variants had a QD value of less than 2. The SNP detection results were annotated using Annovar [23] software.

### 2.7. Genome-Wide Association Analysis

Case control was performed using PLINK v. 1.09, comparing allele frequencies between cases and controls using the chi-square test, a *p*-value with a correlation statistic that indicates whether the observed difference is significant or not. After genotyping, a total of 22988506 SNPs were obtained. Bonferroni correction was used in this study; the suggestive association significance threshold was *p* < 0.05 (*p* < 5.87 × 1.0 × 10^−8^) = (*p* < 0.05/(22988506/27)) at the chromosome-wide level. The significant association indicated that the chromosome-wide level association corresponded to a *p*-value less than 1.0 × 10^−3^ [24]. The association result and significant SNPs were visualized in a Manhattan plot with a threshold line. The Manhattan function was plotted with R v. 3.6.0 [25].

### 2.8. GO and KEGG Enrichment Analysis

Based on the hypergeometric distribution, GO [26] and KEGG [27] pathway enrichment analysis of differential genes were performed to screen the significantly enriched term using R (v 3.2.0). R (v 3.2.0) was used to draw the significant enrichment term’s column, chord, and bubble diagram. Between them, GO level2 was the secondary classification of GO.

### 2.9. Protein Interaction Network Analysis

The interaction relationship in the STRING [28] protein interaction database was used to analyze the differential gene protein interaction network. The differential gene collection was extracted from the database, and the interaction relationship network diagram was constructed by cytoscape [29]. In this study, we performed protein network interactions with proteins related to bacterial infectious diseases, and selected crucial essential node proteins in the entire interaction network.

## 3. Results

### 3.1. Quality Control of whole Genome Sequencing Data of Antagonistic and Susceptible Hu Sheep Lambs

Genome sequencing of 12 blood samples from Hu sheep lambs was completed and a total of 435 Gb of clean reads with high sequencing quality (Q20 ≥ 97.13%, Q30 ≥ 91.80%) were obtained. The comparison rate of all samples was in the range of 97.80–98.30%; the specific quality control results are shown in Table 1. All detected mutations were integrated into a Circos diagram to visualize genomic data, including the density distribution of genomic SNPs on each chromosome (Figure S1), and reference genome alignment data (Figure S2). 

### 3.2. Genome-Wide Association Analysis

**Figure 1:**
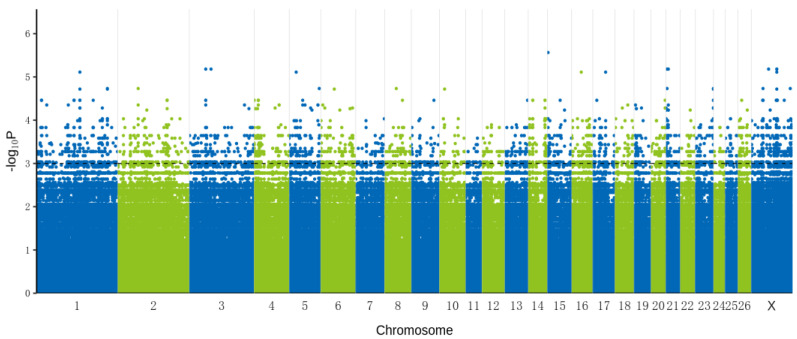
Manhattan plot showing SNPs on sheep chromosomes associated with Hu sheep lambs’ ability to resist *E. coli* F17. The black dashed line corresponds to a suggestive chromosome-wide threshold of 1.0 × 10^−3^.

An association analysis was performed on 22988506 SNPs distributed in 27 chromosomes via whole-genome sequencing analysis of antagonistic and susceptible Hu sheep lamb blood DNA (Figure 1). There were a total of 6868 significant SNPs (*p <* 1.0 × 10^−3^) that are annotated to 466 genes (Table S1).

### 3.3. GO and KEGG Enrichment Analysis

**Figure 2:**
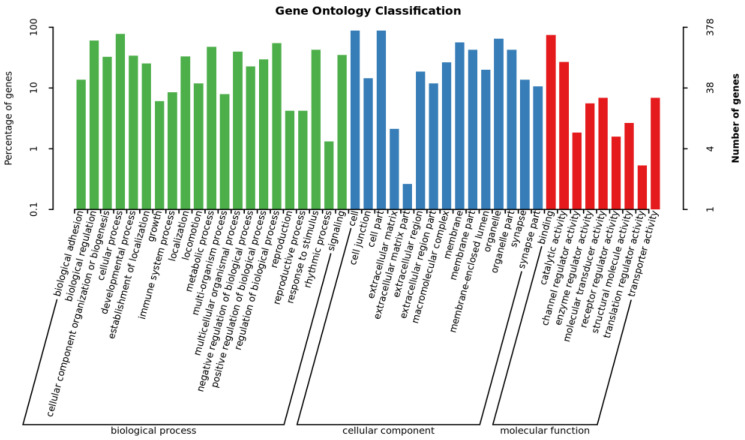
GO terms (gene ontology categories identified in genes with significant SNPs). Note: horizontal coordinates—enriched GO entries; left vertical coordinates—percentage of genes; right vertical coordinates—number of genes.

**Figure 3:**
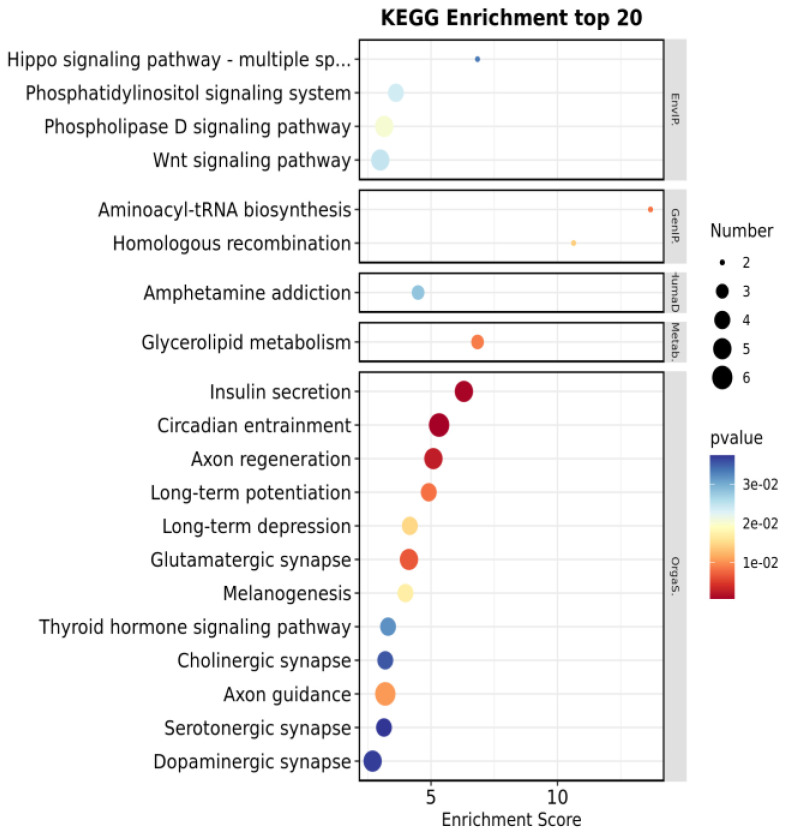
KEGG terms top 20. Note: horizontal scale—enriched scores; vertical scale—enriched entries.

Genome-wide association analysis was used to select SNPs associated at the chromosome level (*p* < 1.0 × 10^−3^); there were 466 genes with significant SNPs for GO and KEGG enrichment analysis. The GO level2 (Figure 2 and Table S2) enrichment results show a total of 45 GO level2 entries, of which GO entries classified as biological processes had the most significant number of GO entries with 21 entries, followed by cell composition with 15 entries. Molecular function ontology had the fewest entries with 9 enrichment items. Biological processes associated with bacterial infections included biological adhesion, immune system process, cell junction, and receptor regulator activity. The KEGG enrichment results (Figure 3 and Table S3) listed the top 20 items (*p <* 0.05) with significant enrichment, among them circadian entrainment, insulin secretion, the Wnt signaling pathway, the thyroid hormone signaling pathway, and the Hippo signaling pathway. Multiple species may be associated with bacterial infectious diseases. 

### 3.4. Protein Interaction Network Analysis

**Figure 4:**
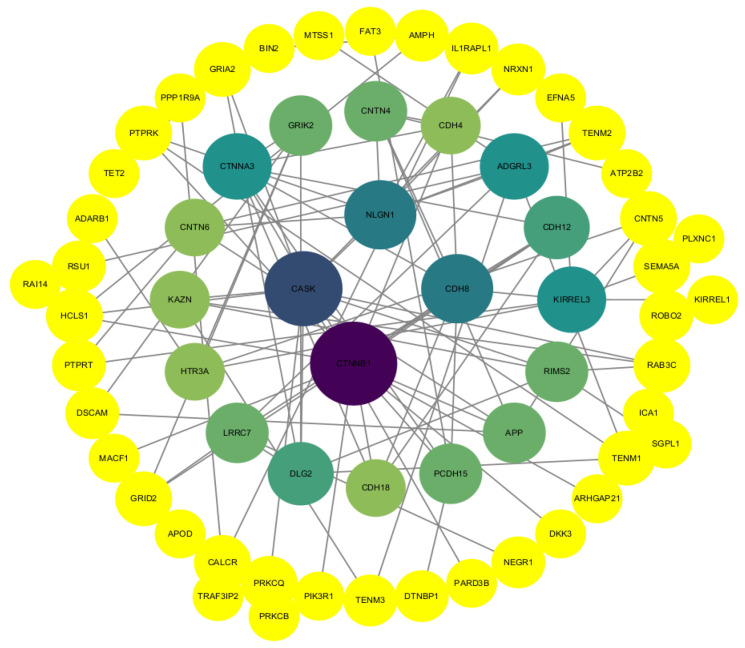
GO-enriched protein interaction network. Nodal protein weights are expressed in colour shades and shape sizes, with darker colours and larger bodies indicating higher weights.

**Figure 5:**
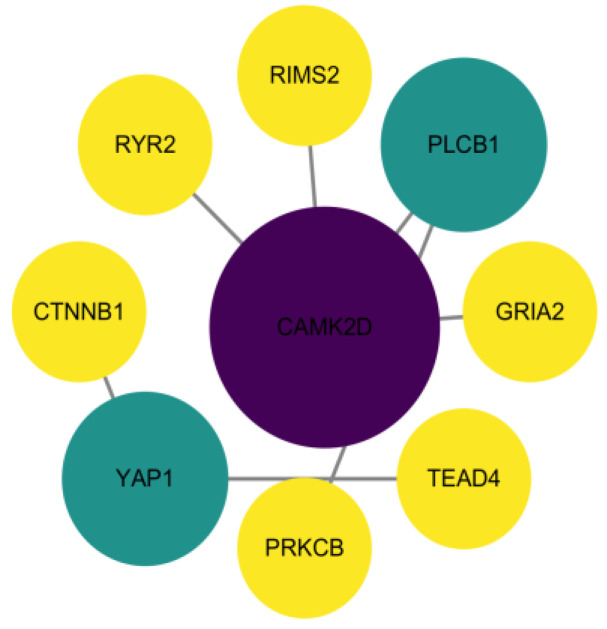
KEGG-enriched protein interaction network. Nodal protein weights are expressed in colour shades and shape sizes, with darker colours and larger bodies indicating higher weights.

The GO-enriched protein interaction network (Figure 4) showed that a total of 62 node proteins interacted with biological adhesion, immune system processes, cell junctions, and receptor regulator activity related to bacterial infections. Candidate proteins CTNNB1, CDH8, APOD, HCLS1, Tet2, MTSS1 were screened. The KEGG protein interaction network (Figure 5) showed multiple species signaling pathways are associated with bacterial infectious diseases: circadian entrainment, insulin secretion, the Wnt signaling pathway, the thyroid hormone signaling pathway, and the Hippo signaling pathway. There were a total of 9 node proteins in the protein interaction network, the candidate gene YAP1 was screened out.

## 4. Discussion

In this study, an association analysis was performed on 22988506 SNPs distributed in 27 chromosomes. There were 466 genes with significant SNPs. GO and KEGG enrichment analysis of genes with differential SNPs was performed. The biological processes associated with bacterial infections in GO enrichment analysis included biological adhesion, immune system processes, cell junctions, and receptor regulator activity. Candidate proteins CTNNB1, CDH8, APOD, HCLS1, Tet2, MTSS1 were screened through the protein interaction network. In the KEGG enrichment analysis, five distinct signaling pathways were involved in the regulation of bacterial infectious diseases, namely circadian entrainment, insulin secretion, the Wnt signaling pathway, the thyroid hormone signaling pathway, and the Hippo signaling pathway—multiple species signaling pathways. The candidate gene YAP1 was screened through the protein interaction network. Preliminary investigations showed that SNPs on the CTNNB1, CDH8, APOD, HCLS1, Tet2, MTSS1 and YAP1 genes may be associated with *E. coli* F17 antagonistic and susceptible Hu sheep lambs.

In GO enrichment analysis and protein–protein interaction network analysis, CTNNB1 (catenin beta 1), an important node protein, is a proto-oncogene encoding β-Catenin protein and a key effector molecule downstream of the Wnt signaling pathway. The function of the Wnt/β-catenin signaling pathway is more about affecting cell proliferation, polarity and tissue homeostasis, which have a variety of phenotypic regulatory functions in signal transduction. The organism must thus maintain normal Wnt/β-catenin signaling, due to the imbalance caused by different external or internal stimuli which change cell proliferation, cause apoptosis and inflammation, etc. In the process of infection with Salmonella typhi, *Salmonella enteritidis*, and *Escherichia coli*, for example, Wnt/β-catenin is an important target of several virulence factors produced by such bacteria [30]. Clostridioides difficile infection is an important pathogenic microorganism that causes diarrhea in elderly people hospitalized for a long time. TcdB is an exotoxin of Clostridioides difficile, which induces macrophage inflammation and epithelial damage, and destroys the normal function of lysosomes in macrophages. TcdB inhibits CTNNB1/β-catenin activity and lysosomal acidification in macrophages; the resulting lysosomal dysfunction affects the inflammatory response and causes tissue damage through autophagy [31]. It has been reported that mutations at the upper site of CTNNB1 are associated with tuberculosis caused by a conjugated divergent bacterium; this study also showed that CTNNB1 can affect host susceptibility to a conjugated divergent bacterium and this can be used as a marker gene to identify tuberculosis [32]. CDH8 is a classic cadherin which has been studied in bacterial infectious diseases. For example, an endemic wild boar population was divided into a Mycobacterium tuberculosis-infected group and an uninfected group, and genome-wide association analysis screened CDH8 as a candidate gene for host genetic susceptibility to tuberculosis caused by divergent bacteria [33]. More research on CDH8 has shown that mutations in the CDH8 gene lead to cerebellar hypoplasia and motor dysfunction, also an essential factor in autism [34,35,36]. In order to clarify the genetic background of pig susceptibility to ETEC *E. coli* F4ab/ac and establish a haplotype map of candidate regions, experimental materials were taken from European wild boar and Swedish Large White pigs, and the susceptibility of pigs to F4ab/ac was classified by adhesion experiments. The candidate regions were also screened for polymorphisms, including the presence of the APOD gene, which may be related to the susceptibility of *E. coli* F4ab/ac [37]. Brucellosis is a major zoonotic disease worldwide and poses a major threat to human safety. The literature reports that there is a genetic association between PTPRT gene polymorphisms and goats’ susceptibility to Brucella [38]. Kostmann’s disease is a rare autosomal recessive form of severe congenital neutropenia, characterized by the cessation of promyelocyte/myelocyte maturation in the bone marrow caused by homozygous mutations in the X1 gene encoding the mitochondrial protein HCLS1 [39], resulting in severe recurrent bacterial infections in early infancy. The Tet2 gene is involved in the immune response of bone marrow cells and lymphocytes to infections of respiratory epithelial cells by Pseudomonas aeruginosa, affecting the regulation of host defence against it [40]. In addition, the TET2 gene also affects the host’s resistance to tuberculosis, the specific mechanism being that the binding of Tet2 to NF-κB induces the demethylation of TNF, where NF-κB is an important factor affecting the phenotypes of cell proliferation, apoptosis and inflammation. It is also an indispensable signal transduction pathway in the body; it affects the release of downstream TNF and then changes the host’s immune response. It can be used as a new host immunization strategy against tuberculosis [41]. Staphylococcus aureus EDIN toxin can induce endothelial cell torn macrophyllum (TEM) tunneling, thereby destroying the host endothelial barrier and promoting bacterial transmission. In the loss of MIM (MTSS1) in the transfer of I-BAR domain proteins, the toxin can sense the occurrence of TEM, and finally induce TEM, so that MTSS1 can change the integrity of the host endothelial barrier and affect the invasion of toxins [42]. These studies revealed that CTNNB1, CDH8, APOD, PTPRT, HCLS1, Tet2 and MTSS1 genes can change the host’s resistance to bacterial infections. In this study, these genes are also annotated by whole genome sequencing and association analysis in antagonistic and susceptible Hu lambs; the susceptibility of Hu lambs to *E. coli* F17 may be related to these genes.

In the KEGG enrichment analysis, circadian entrainment is associated with innate immunity. The gut microbiota produces circadian rhythms and the rhythmic expression of antimicrobial proteins is driven by the daily rhythm of epithelial attachment by segmented filamentous bacteria (SFB) (a member of the mouse gut microbiota), activating immune circuits involving group 3 innate lymphoid cells. This circuit triggers oscillations of STAT3 expression and activation in epithelial cells, producing rhythmic antimicrobial protein expression that affects resistance to Salmonella typhimurium infection [43]. Perception of microbial ligands by macrophages triggers an increase in glucose metabolism, which provides energy to support antimicrobial inflammation and cytokine production and results in insulin secretion which regulates glucose homeostasis [44]. The Wnt signaling pathway can regulate intestinal epithelial cell proliferation, maintain the intestinal mucosal barrier’s integrity, induce Paneth cell differentiation, and increase the expression of antimicrobial peptides [45]. In addition, Wnt signaling pathway involvement plays a key role in the tug-of-war between hosts and pathogens [46]. The thyroid hormone signaling pathway is associated with Neisseria meningitidis. Thyroxine supplementation in mice infected with Neisseria meningitidis enhances bacterial clearance, reduces inflammation, and promotes survival [47]. In a study of mastitis caused by Staphylococcus aureus infection, the Hippo signaling pathway was activated in a transcriptome analysis of Staphylococcus aureus infection and uninfected MAC-T cells [48]. In the interaction of five gene protein networks that regulate bacterial infectious disease signaling, the transcription factor YAP-1/YAP is activated when Pseudomonas aeruginosa infects the intestinal barrier of worms; the deletion of YAP-1 significantly reduces the survival rate of worms and increases the accumulation of bacteria in the host, which deploys pathogen defenses when it recognizes the loss of the intestinal epithelial barrier [49]. Another study also pointed out that YAP affects pathogen infection in the pulmonary epithelial barrier, and that in order to maintain this barrier, the regeneration of alveolar epithelium plays an essential role in lung damage caused by infectious pathogens. In the lung inflammation of Streptococcus pneumoniae infection, mice lacking Yap/Taz in alveolar epithelial type II cells exhibited a prolonged lung inflammatory response and delayed alveolar epithelial regeneration, affecting the reconstruction of the lung epithelial barrier [50]. In KEGG enrichment analysis and protein–protein interaction network analysis, this paper describes the functions of circadian entrainment, insulin secretion, the Wnt signaling pathway, the thyroid hormone signaling pathway, and the Hippo signaling pathway—multiple species signaling pathways. The candidate gene YAP1 was screened out.

Despite the results of whole genome association analysis, via GO and KEGG enrichment analysis, and protein interaction network analysis, we have predicted the genetic loci and candidate genes related to the resistance of Hu sheep to *E. coli* F17. However, there are still some shortcomings that need to be verified by in vivo and in vitro functional experiments to verify the correlation between candidate gene functions and genetic loci. This is therefore our next line of research.

In recent years, as the demand for sheep has increased and the scale of sheep production has expanded, the likelihood of sheep diseases has also increased. The rapid spread of sheep diseases has led to an increase in the culling of sheep and losses to breeding farms. *Escherichia coli* infections are a major threat to young animals and can be fatal in severe cases [51,52]. Therefore, the breeding of high quality disease-resistant sheep is an important issue, and the use of key genes or candidate sites that influence the occurrence of sheep diseases can improve the economic benefits of breeding enterprises and promote the healthy development of the sheep industry. However, there are few such studies in sheep. There are more studies on sheep economic, growth and development traits, such as the genome-wide association analysis to study SNPs related to body size in Hu sheep, and to screen 5 SNPs related to body height and 4 SNPs related to chest circumference at the chromosomal significance level [53]. Genome-wide association analysis and the phenotype of sheep wool and skin wrinkles have identified variants in several candidate genes [54], as has a genome-wide association analysis to study sheep production and reproductive performance [55]. At present, some genes have been verified by experiments that elucidate their genetic mechanisms, but there are still genes to be discovered and studied in the future. In the last few decades, genome-wide association analysis has provided a large body of reference materials for sheep genetics and breeding, which can identify sites of variation, and key genes and pathways associated with sheep phenotypes, to better understand the genetic mechanisms of the complex forms of sheep. Genome-wide association analysis has been widely used in animal breeding. With the innovation of technology, artificial intelligence, machine learning, and the application of sequencing has enabled more accurate data sequencing results and identification of the more meaningful main genes. At the same time, an increase in sample size provides more accurate predictions. In addition, multi-omics combined analysis is also widely used, and the association analysis of the transcriptome, translation, proteome and metabolome of important trait indicators in sheep can explain problems from multiple directions.

## 5. Conclusions

Whole genome sequencing and whole genome association analysis revealed 466 genes with significant SNPs (*p* < 1.0 × 10^−3^). GO and KEGG enrichment analysis, protein–protein interaction network analysis, and preliminary investigations showed that SNPs on CTNNB1, CDH8, APOD, HCLS1, Tet2, MTSS1 and YAP1 genes may be associated with the antagonism and susceptibility of Hu sheep lambs to *E. coli* F17. This study provides genetic loci and candidate genes for resistance of Hu sheep lambs to *E. coli* F17 infection and provides a genetic basis for breeding disease-resistant sheep.

## Figures and Tables

**Table 1:** Summary of QC results.

Samples	R-R	C-R	C-R-P	R-B	C-B	C-B-P	G-C	>Q20	>Q30
s2S-250	328124474	323466836	98.58%	49.22G	48.38G	98.30%	43.32%	97.56%	93.05%
s2S-936	316593058	311927296	98.53%	47.49G	46.66G	98.26%	43.47%	97.52%	92.96%
s2S-088	280099462	275608400	98.40%	42.01G	41.22G	98.10%	43.24%	97.08%	91.80%
s2S-037	291558274	286959356	98.42%	43.73G	42.89G	98.06%	43.41%	97.30%	92.28%
s2S-341	298812648	293506672	98.22%	44.82G	43.84G	97.80%	43.63%	97.13%	91.87%
s2S-940	292608542	288114728	98.46%	43.89G	43.05G	98.08%	43.58%	97.24%	92.08%
s2A-926	229463262	225940978	98.46%	34.42G	33.80G	98.21%	43.48%	97.55%	93.06%
s2A-965	251932032	248203718	98.52%	37.79G	37.10G	98.17%	43.40%	97.69%	93.38%
s2A-349	253327108	249116262	98.34%	38.00G	37.22G	97.94%	43.76%	97.19%	92.02%
s2A-187	266199676	261823920	98.36%	39.93G	39.13G	98.01%	43.37%	97.14%	91.90%
s2A-982	247628212	243590354	98.37%	37.14G	36.40G	97.99%	43.60%	97.23%	92.22%
s2A-937	229237666	225756938	98.48%	34.39G	33.69G	97.98%	43.09%	97.45%	92.68%

Note: R-R (raw reads), C-R (clean reads), C-R-P (clean reads percent), R-B (raw base), C-B (clean base), C-B-P (clean base percent), G-C (GC content).

## Data Availability

The data presented in this study are openly available in the SRA database, accession number PRJNA996327.

## Supplementary Materials

The following supporting information can be downloaded at: https://www.mdpi.com/article/10.3390/ani14010161/s1. Figure S1: The density of SNPs distributed across chromosomes; Figure S2: Genome-wide coverage of every sample; Table S1: Variant location information; Table S2: GO level 2 enrichment; Table S3: KEGG enrichment.
