# Supplementary figures and images for: A Study of the Resistance of Hu Sheep Lambs to Escherichia coli F17 Based on Whole Genome Sequencing
Source: Animals (Basel). 2024 Jan 3;14(1):161. doi: 10.3390/ani14010161 (PMC10778179; doi:10.3390/ani14010161)

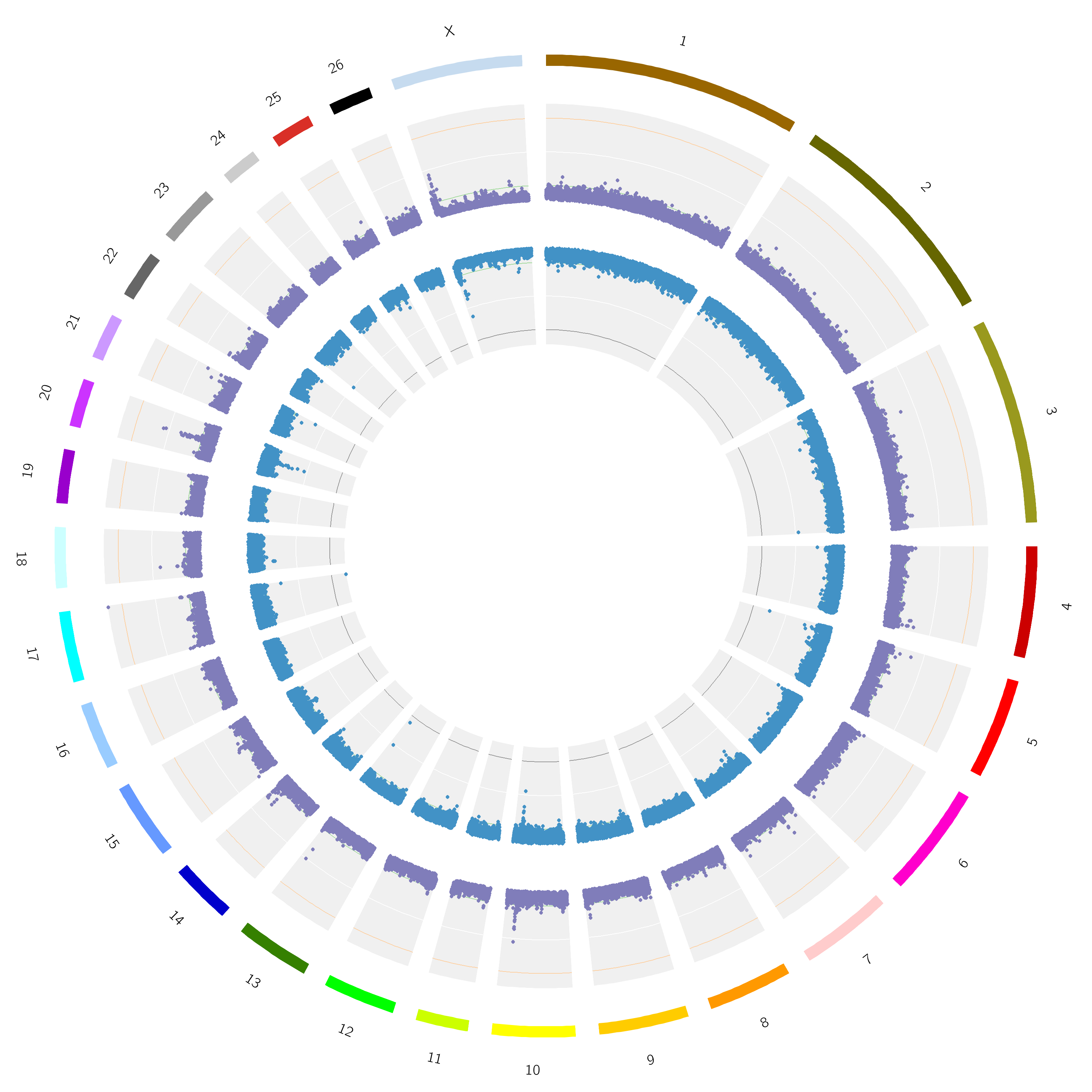

Supplement: Supplementary file 1 [file animals-14-00161-s001.zip › Supplementary Material 1/s2A-187/s2A-187.circos.png]

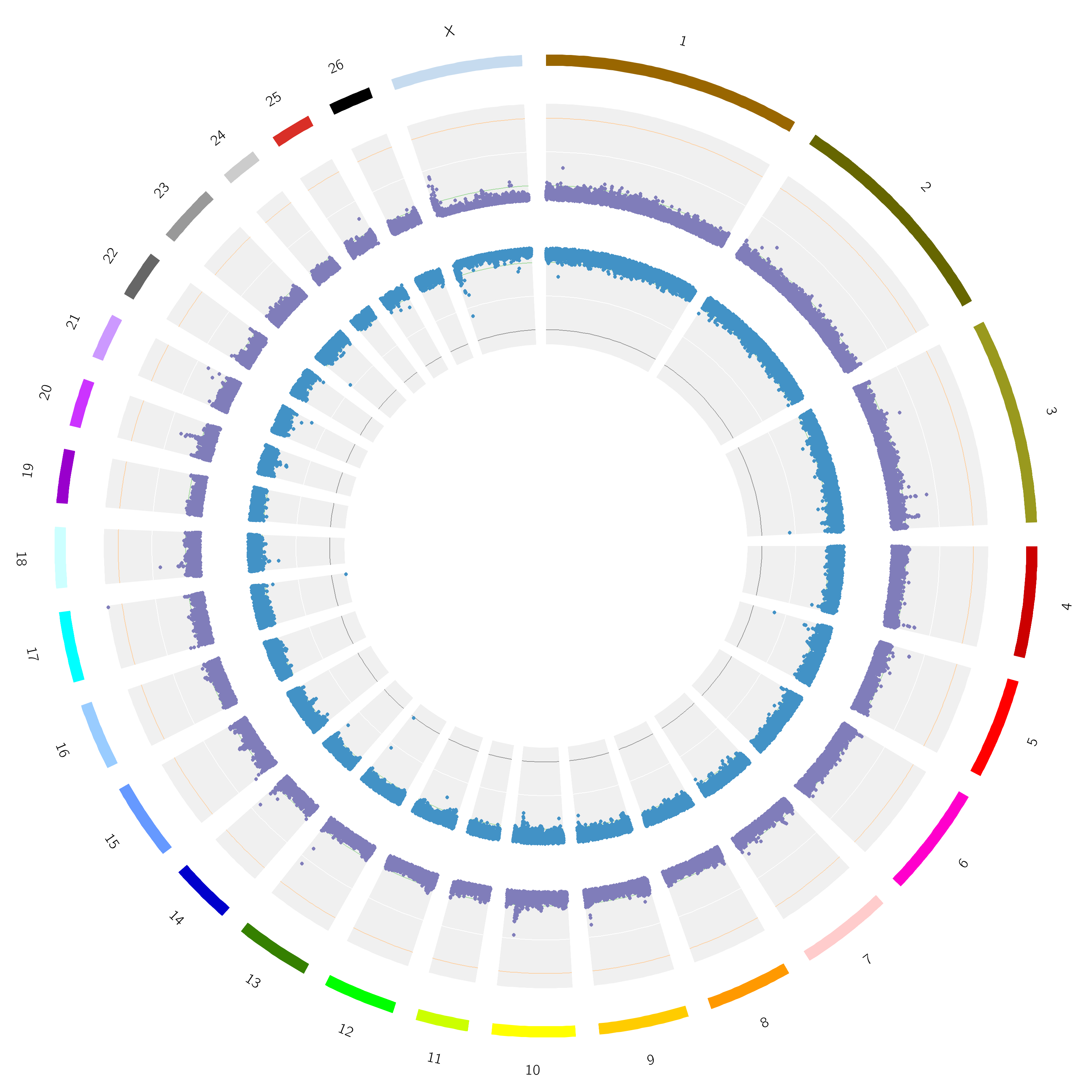

Supplement: Supplementary file 1 [file animals-14-00161-s001.zip › Supplementary Material 1/s2A-349/s2A-349.circos.png]

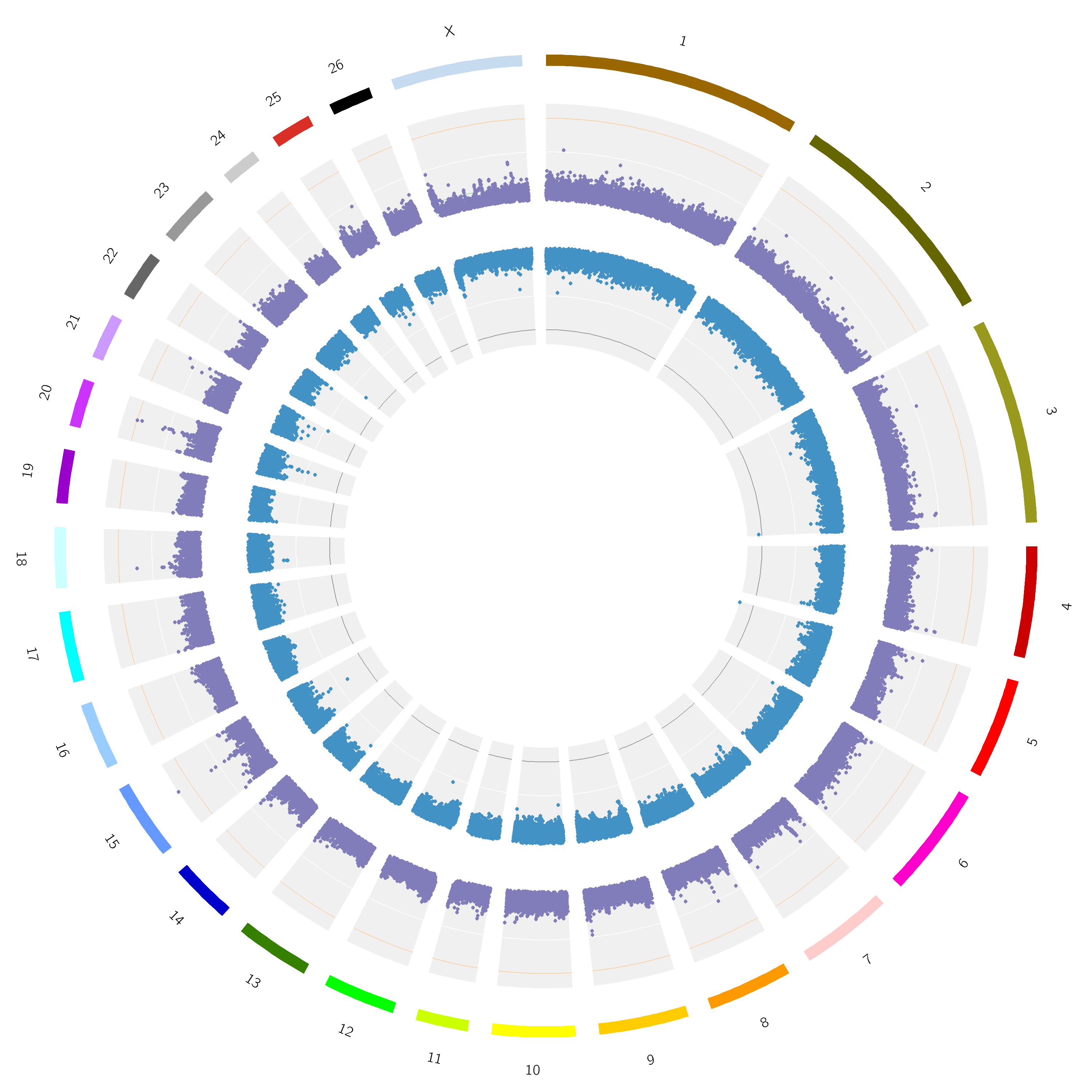

Supplement: Supplementary file 1 [file animals-14-00161-s001.zip › Supplementary Material 1/s2A-926/s2A-926.circos.png]

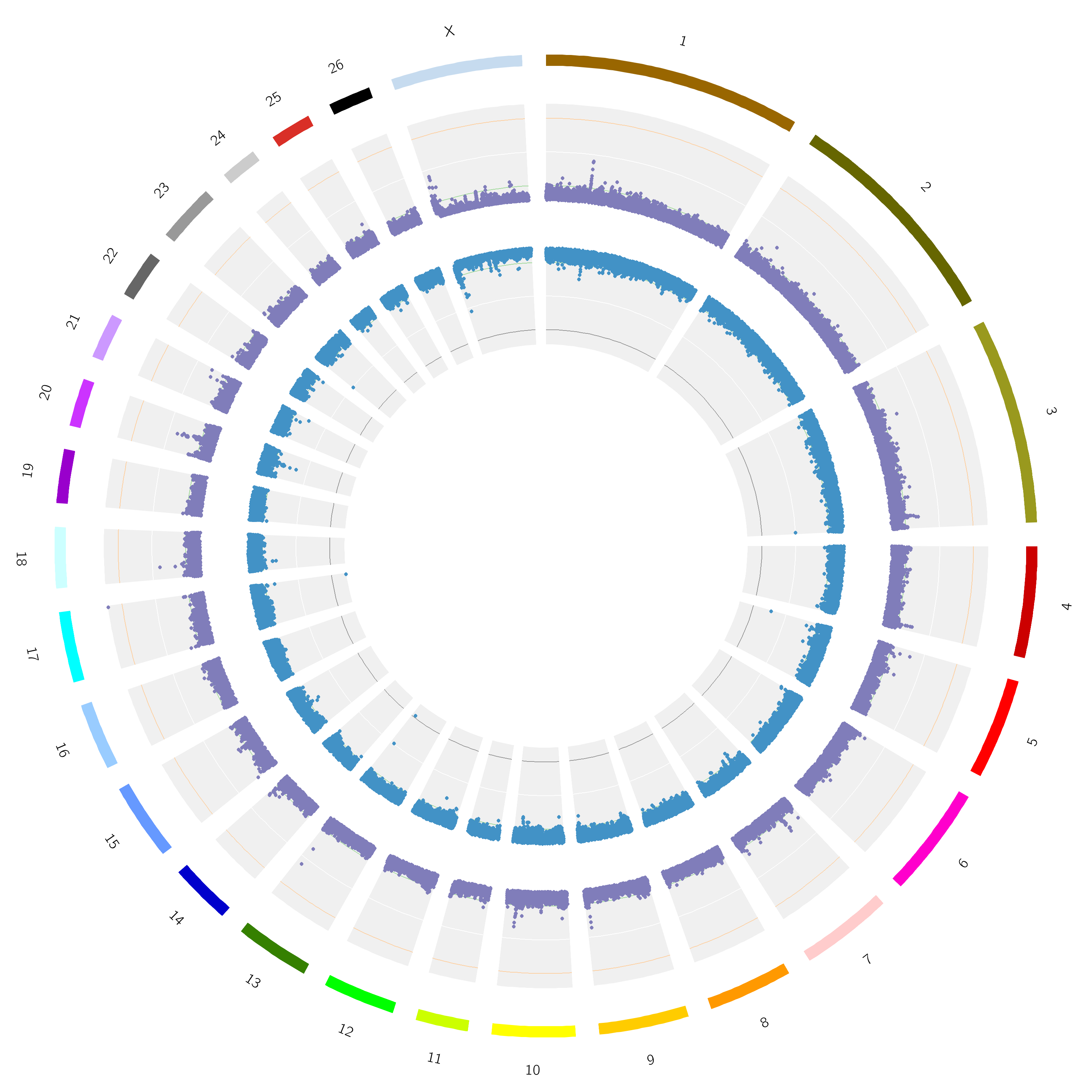

Supplement: Supplementary file 1 [file animals-14-00161-s001.zip › Supplementary Material 1/s2A-937/s2A-937.circos.png]

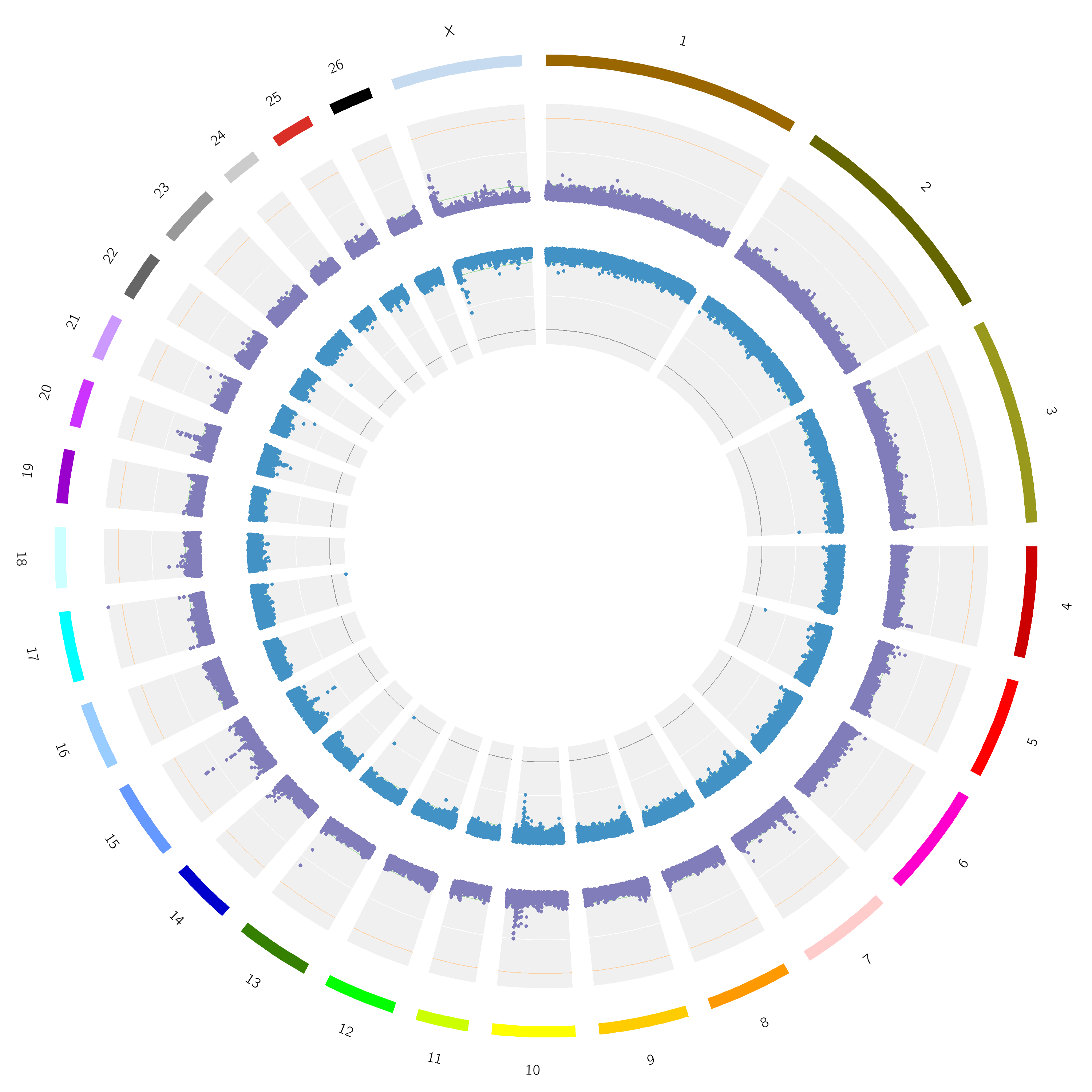

Supplement: Supplementary file 1 [file animals-14-00161-s001.zip › Supplementary Material 1/s2A-965/s2A-965.circos.png]

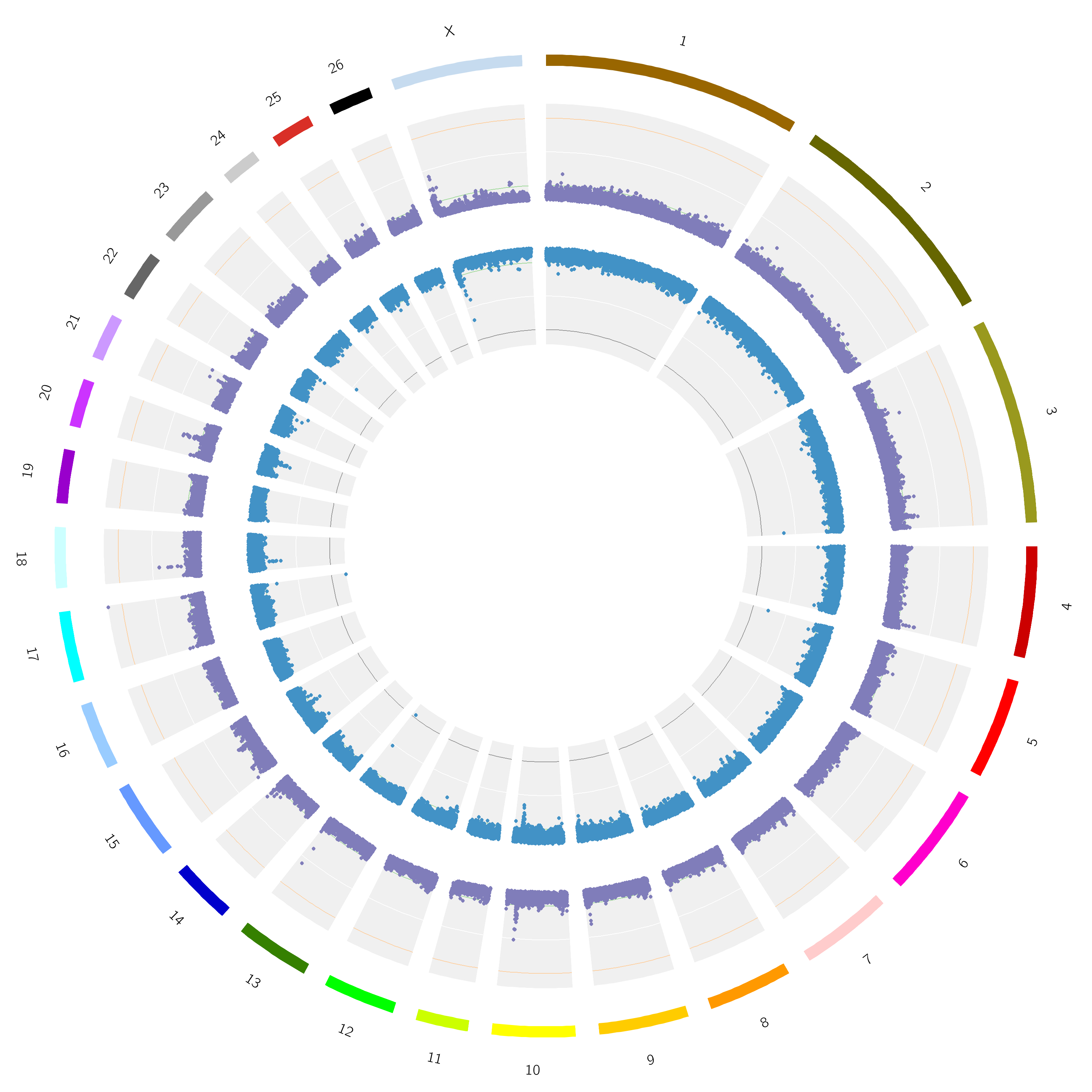

Supplement: Supplementary file 1 [file animals-14-00161-s001.zip › Supplementary Material 1/s2A-982/s2A-982.circos.png]

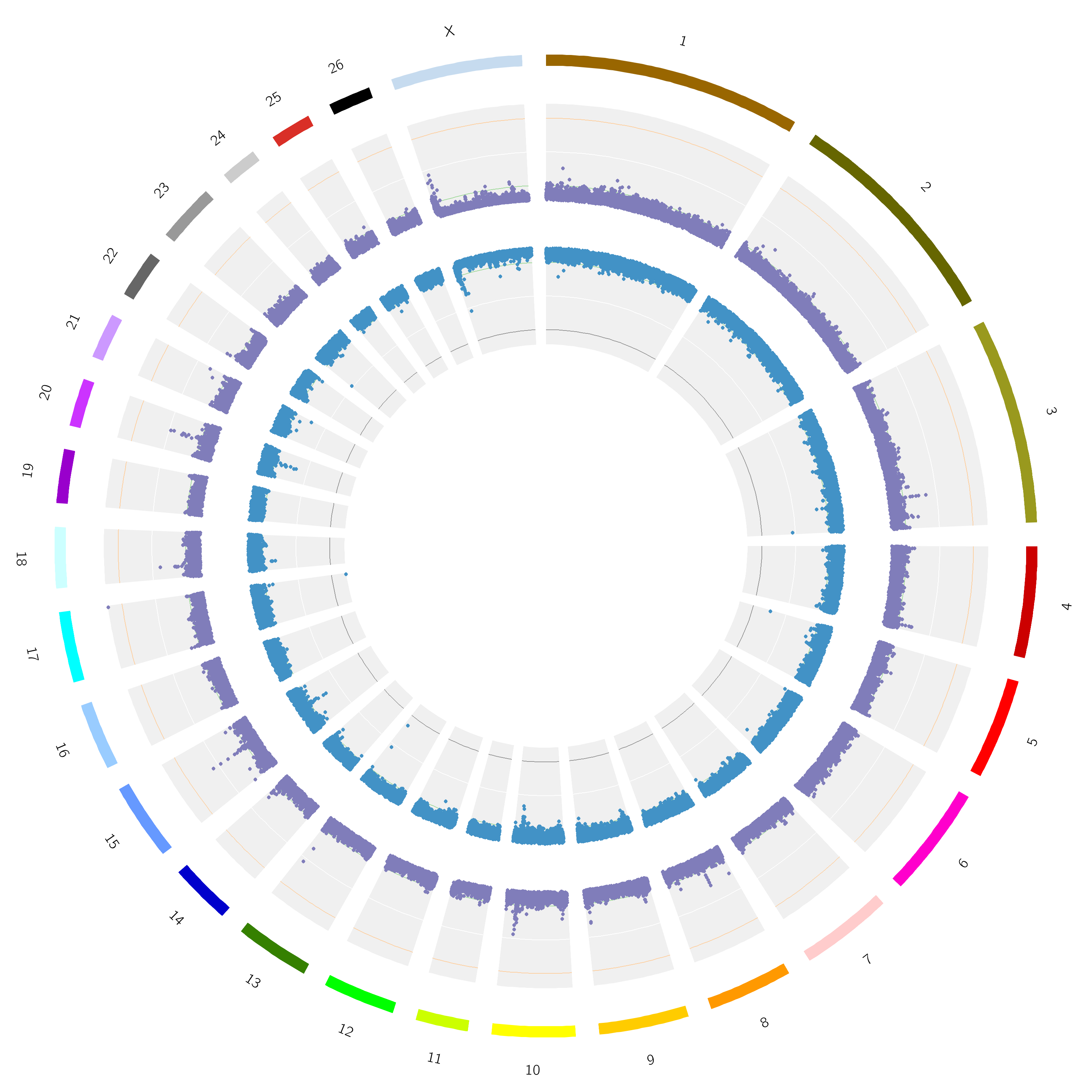

Supplement: Supplementary file 1 [file animals-14-00161-s001.zip › Supplementary Material 1/s2S-037/s2S-037.circos.png]

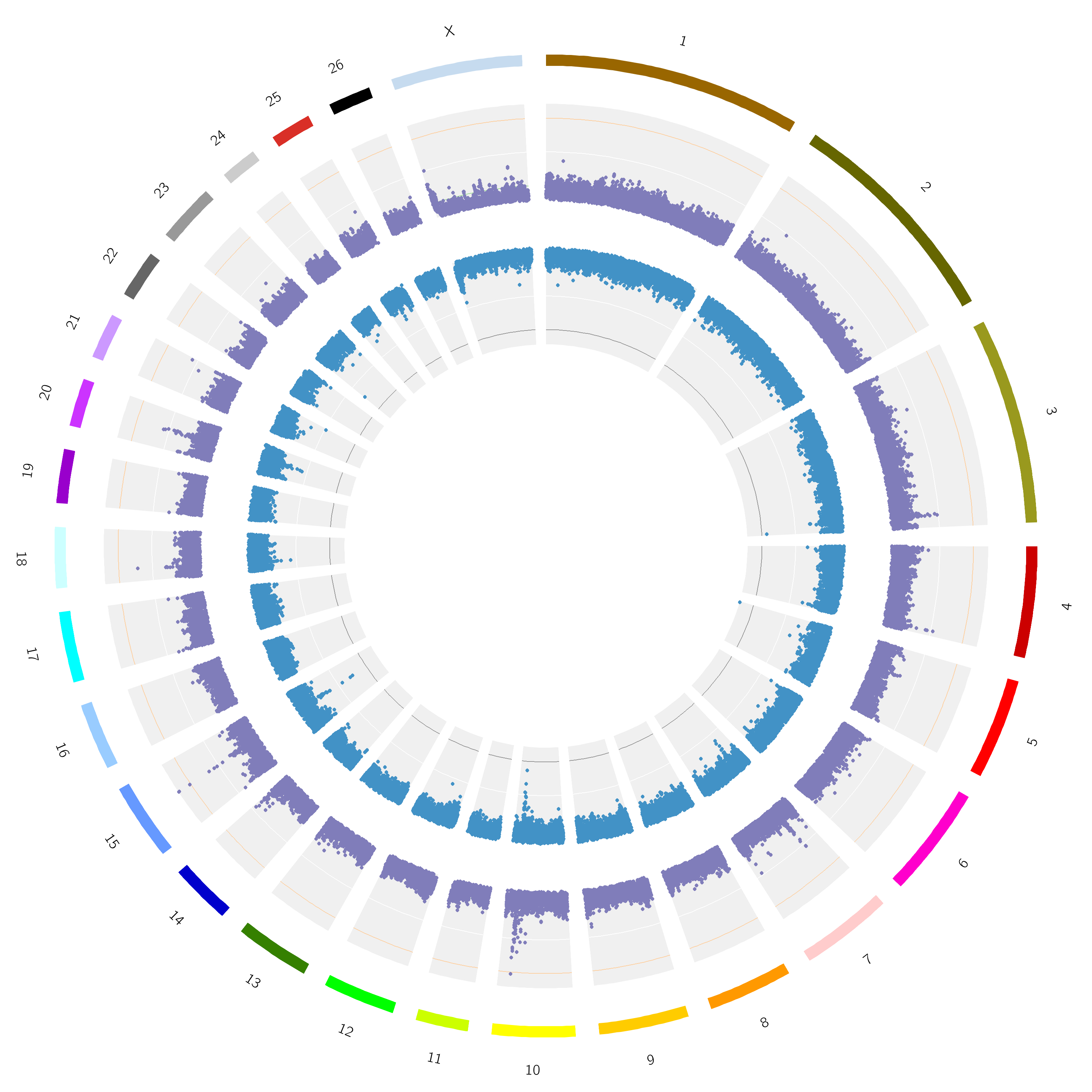

Supplement: Supplementary file 1 [file animals-14-00161-s001.zip › Supplementary Material 1/s2S-088/s2S-088.circos.png]

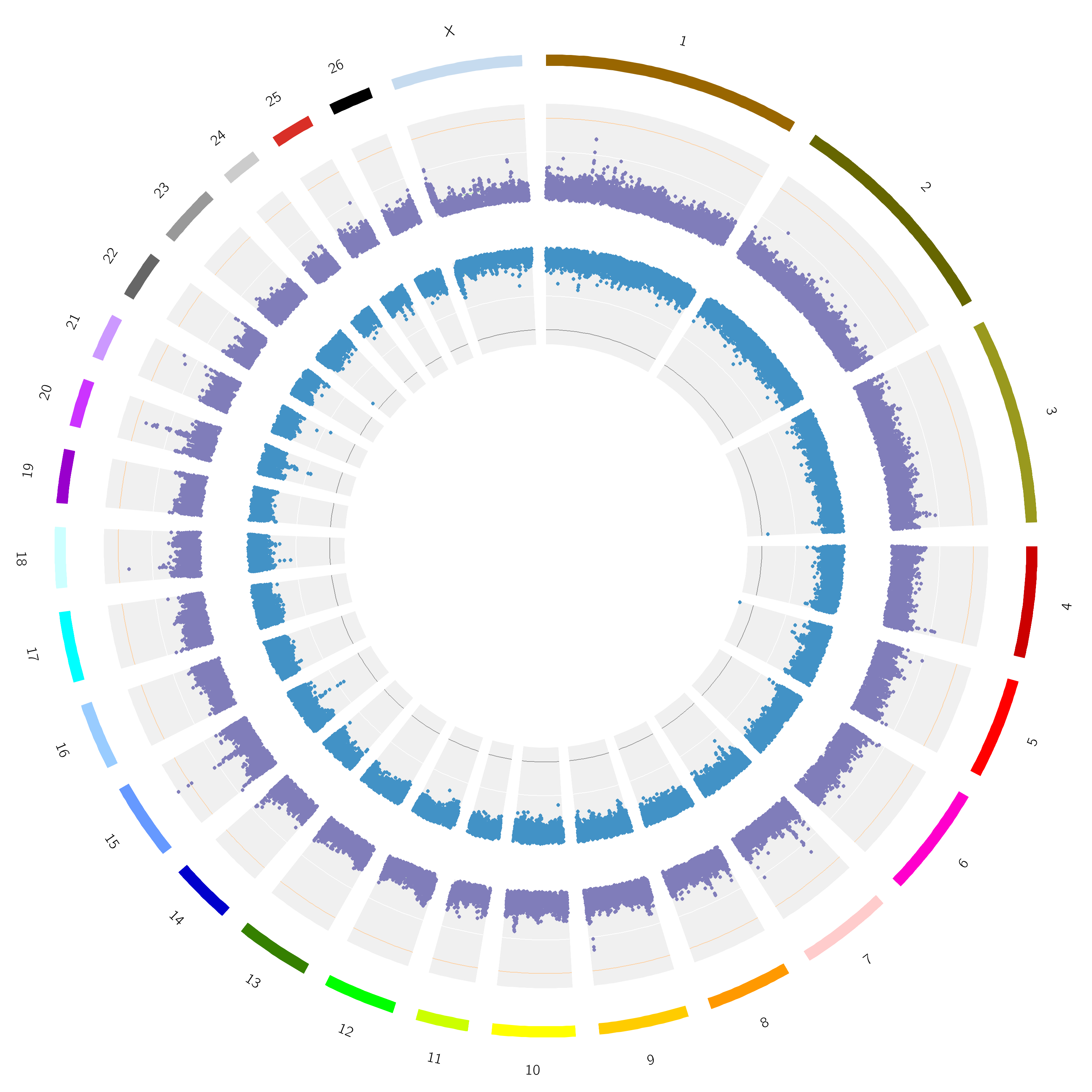

Supplement: Supplementary file 1 [file animals-14-00161-s001.zip › Supplementary Material 1/s2S-250/s2S-250.circos.png]

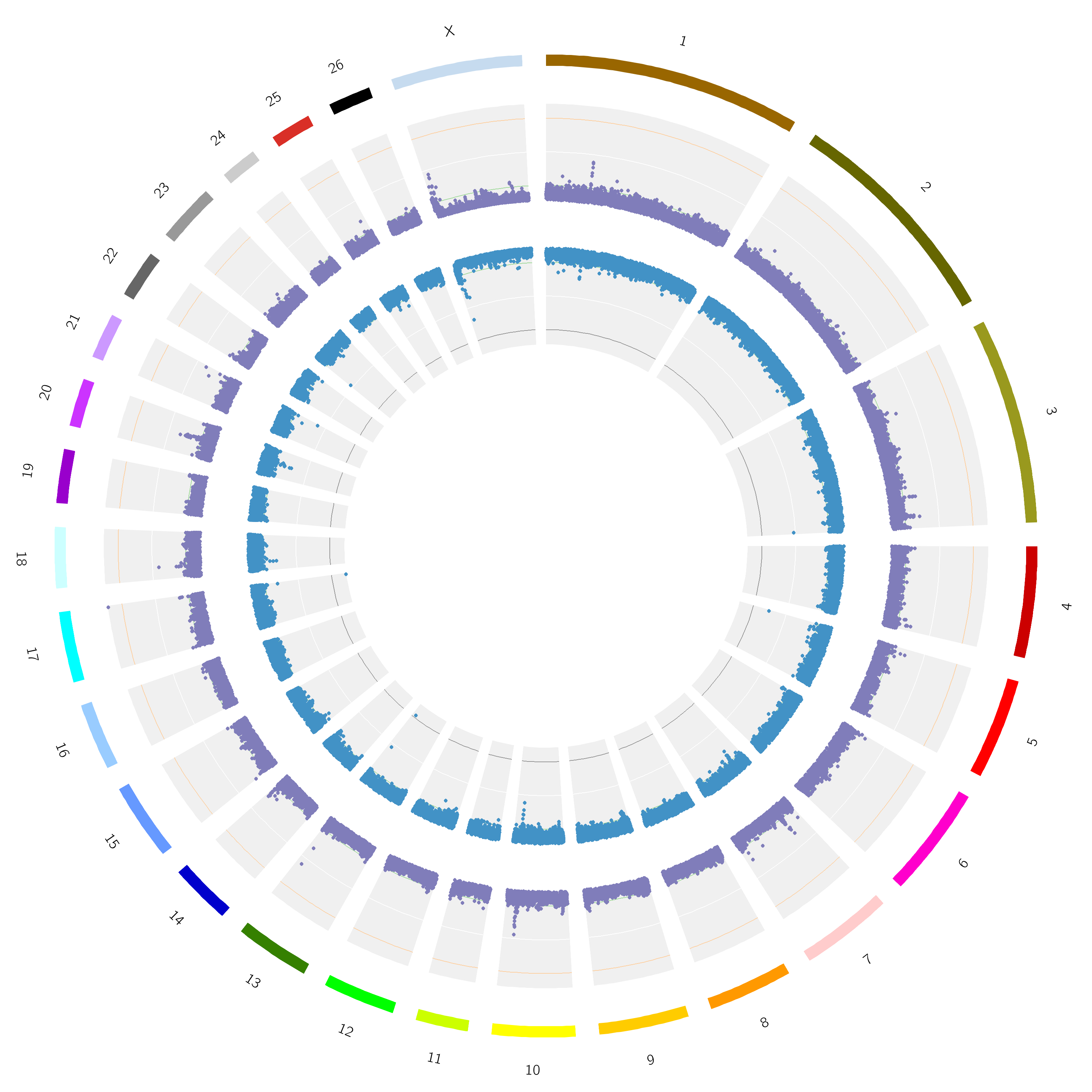

Supplement: Supplementary file 1 [file animals-14-00161-s001.zip › Supplementary Material 1/s2S-341/s2S-341.circos.png]

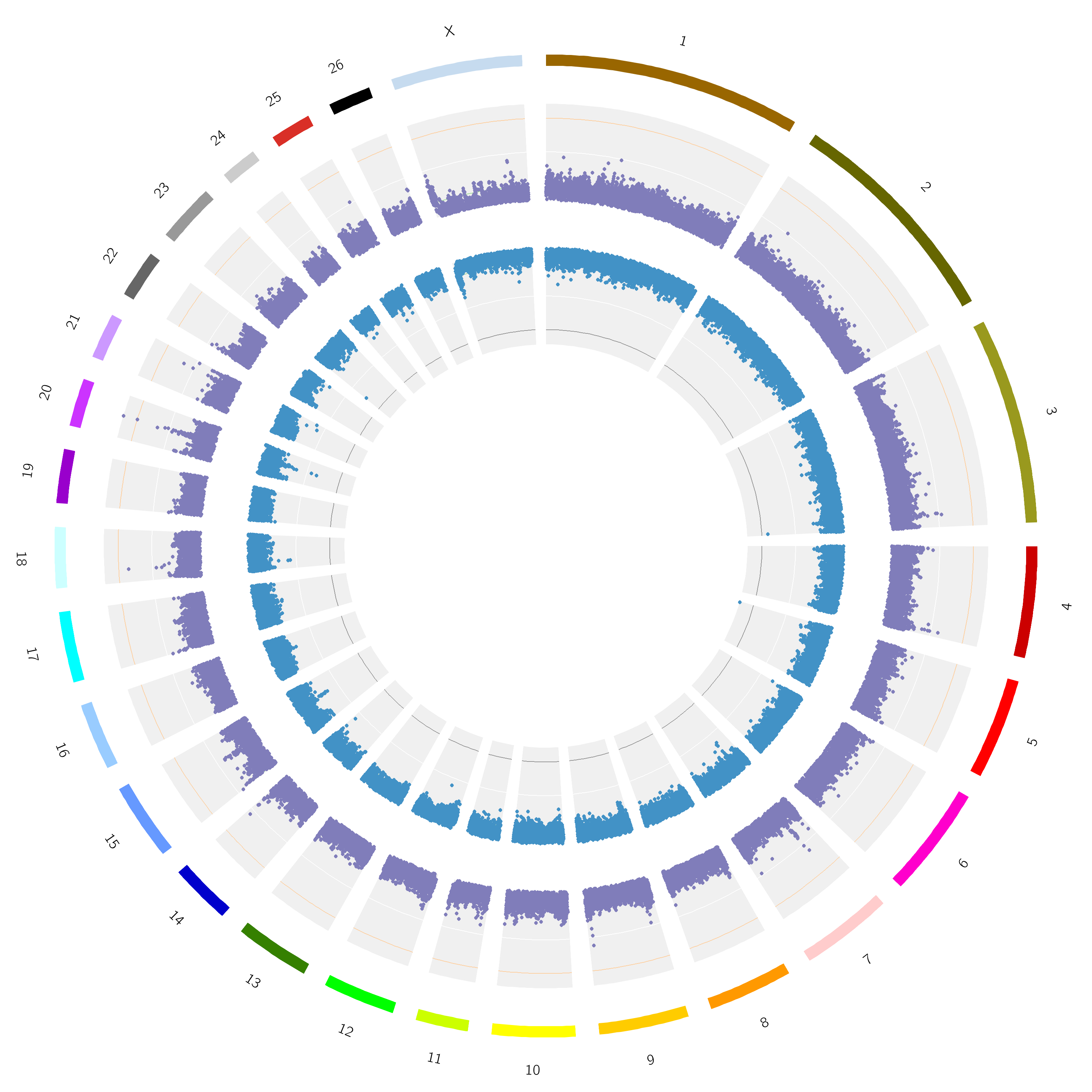

Supplement: Supplementary file 1 [file animals-14-00161-s001.zip › Supplementary Material 1/s2S-936/s2S-936.circos.png]

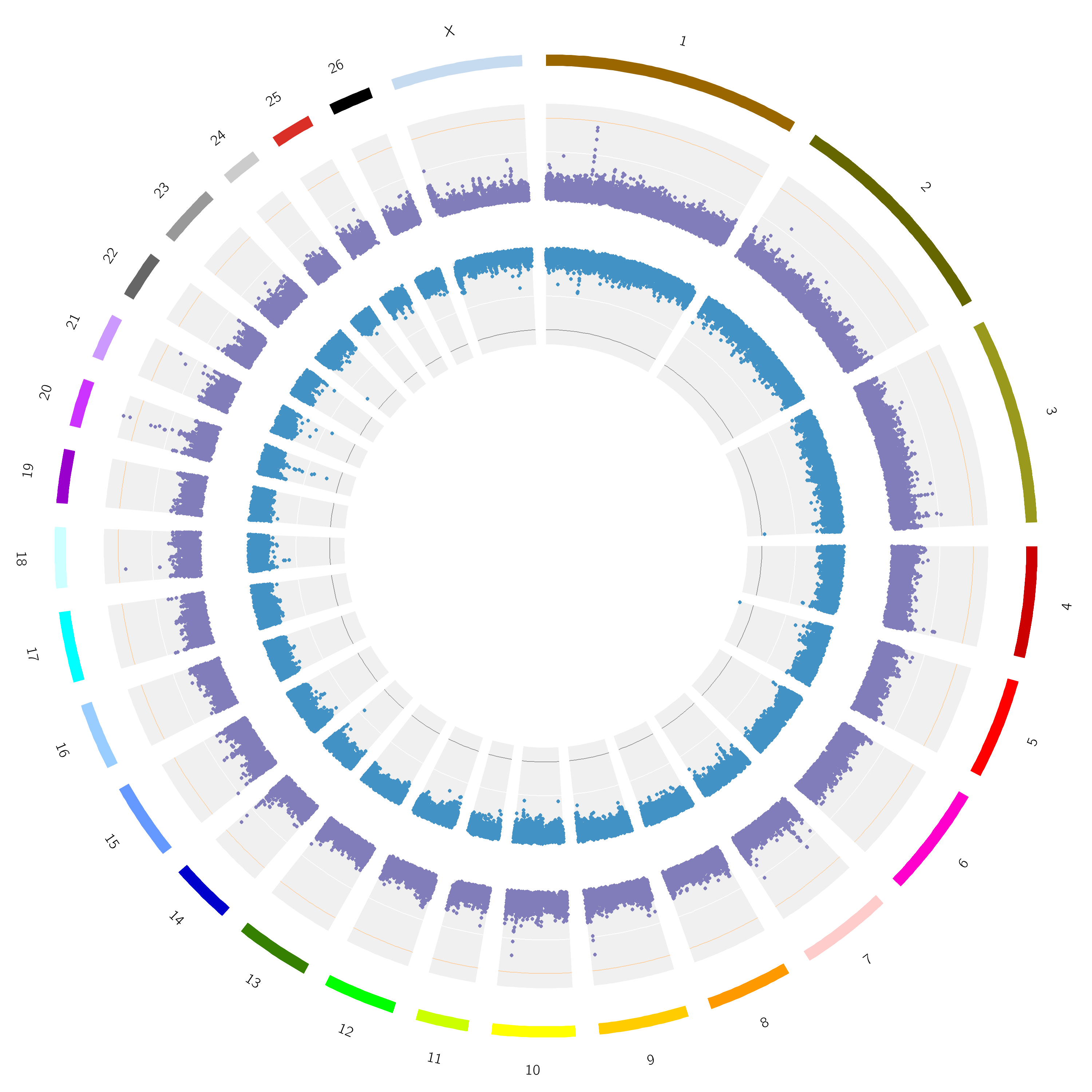

Supplement: Supplementary file 1 [file animals-14-00161-s001.zip › Supplementary Material 1/s2S-940/s2S-940.circos.png]

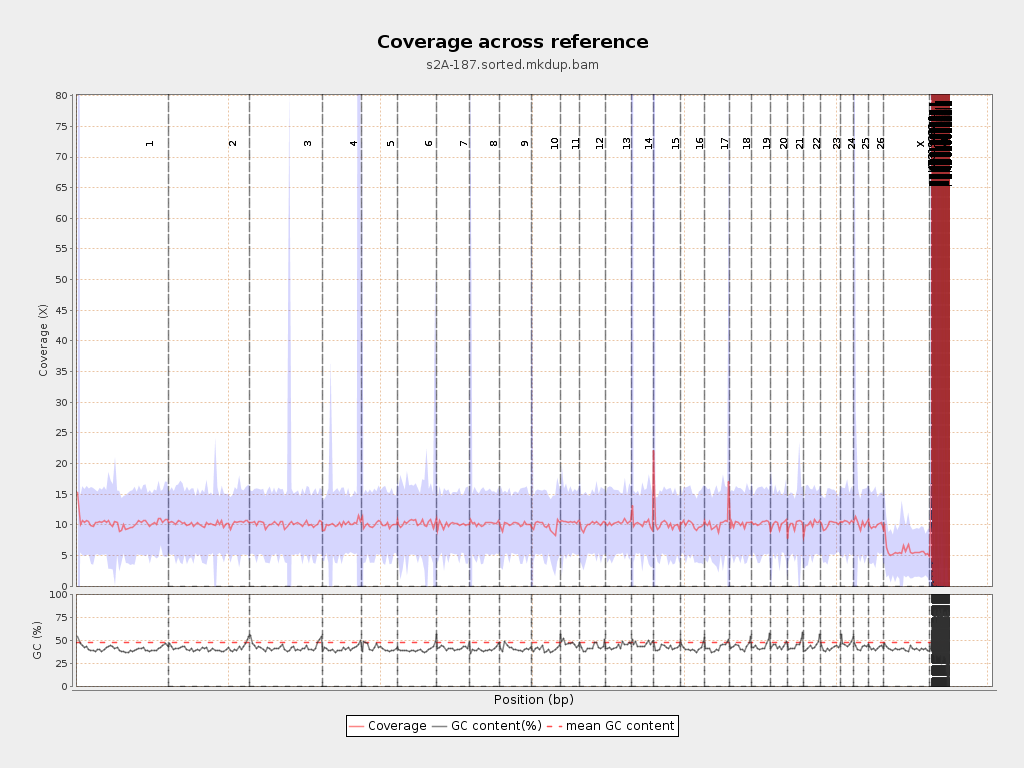

Supplement: Supplementary file 1 [file animals-14-00161-s001.zip › Supplementary Material 2/s2A-187/genome_coverage_across_reference.png]

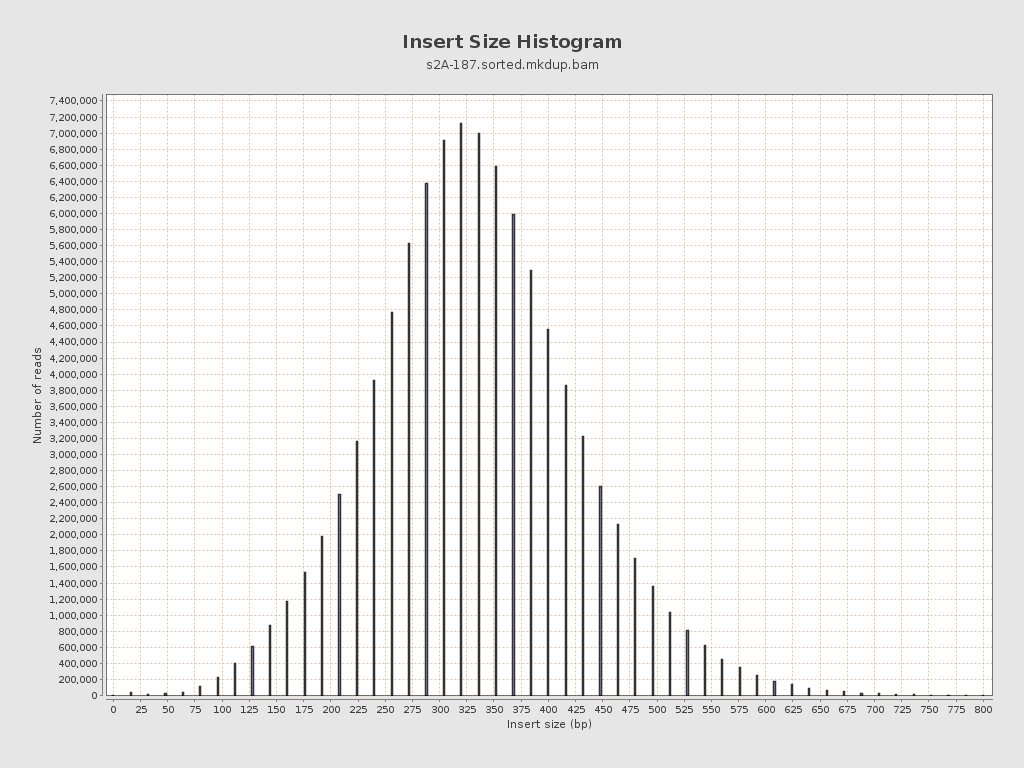

Supplement: Supplementary file 1 [file animals-14-00161-s001.zip › Supplementary Material 2/s2A-187/genome_insert_size_histogram.png]

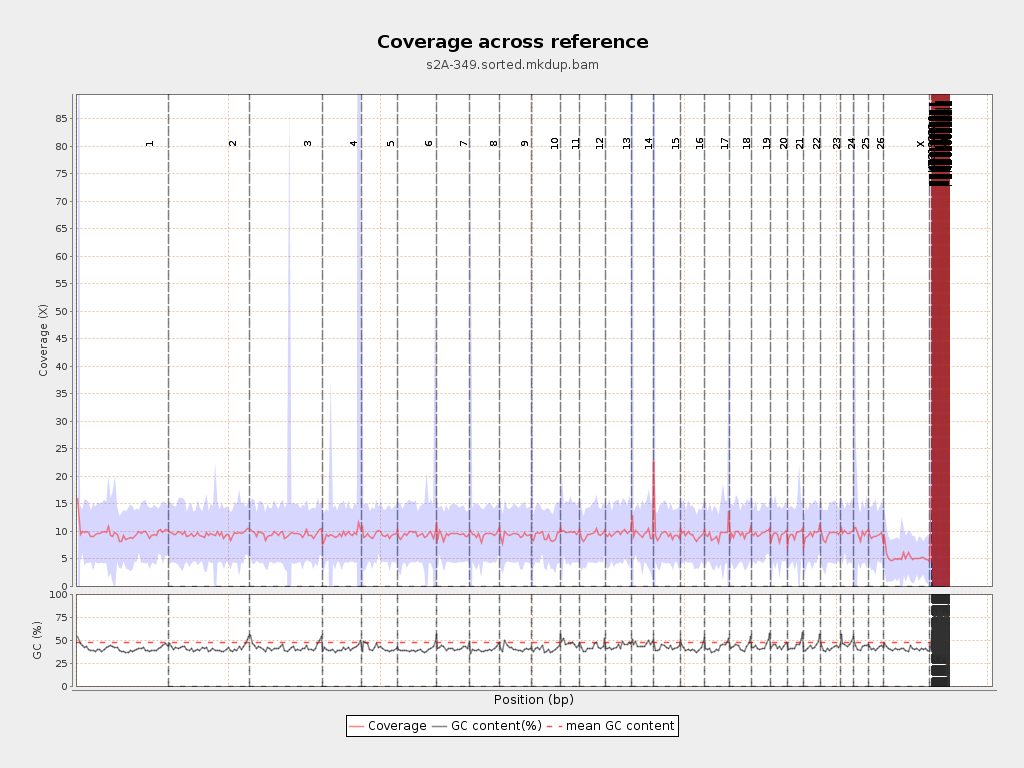

Supplement: Supplementary file 1 [file animals-14-00161-s001.zip › Supplementary Material 2/s2A-349/genome_coverage_across_reference.png]

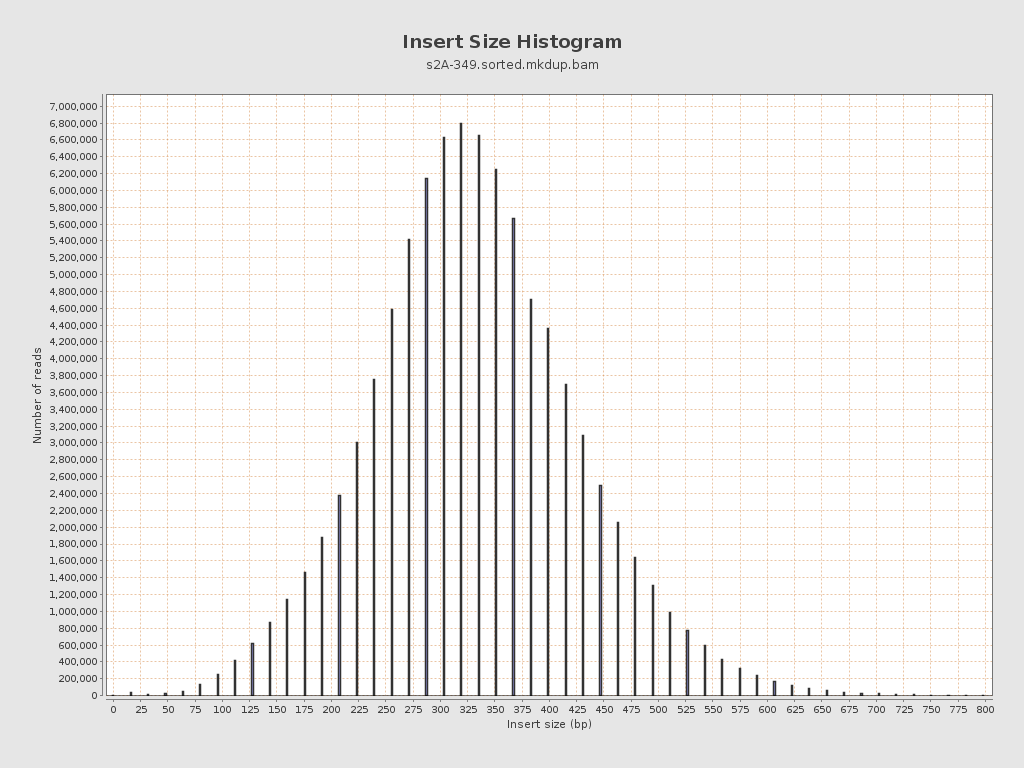

Supplement: Supplementary file 1 [file animals-14-00161-s001.zip › Supplementary Material 2/s2A-349/genome_insert_size_histogram.png]

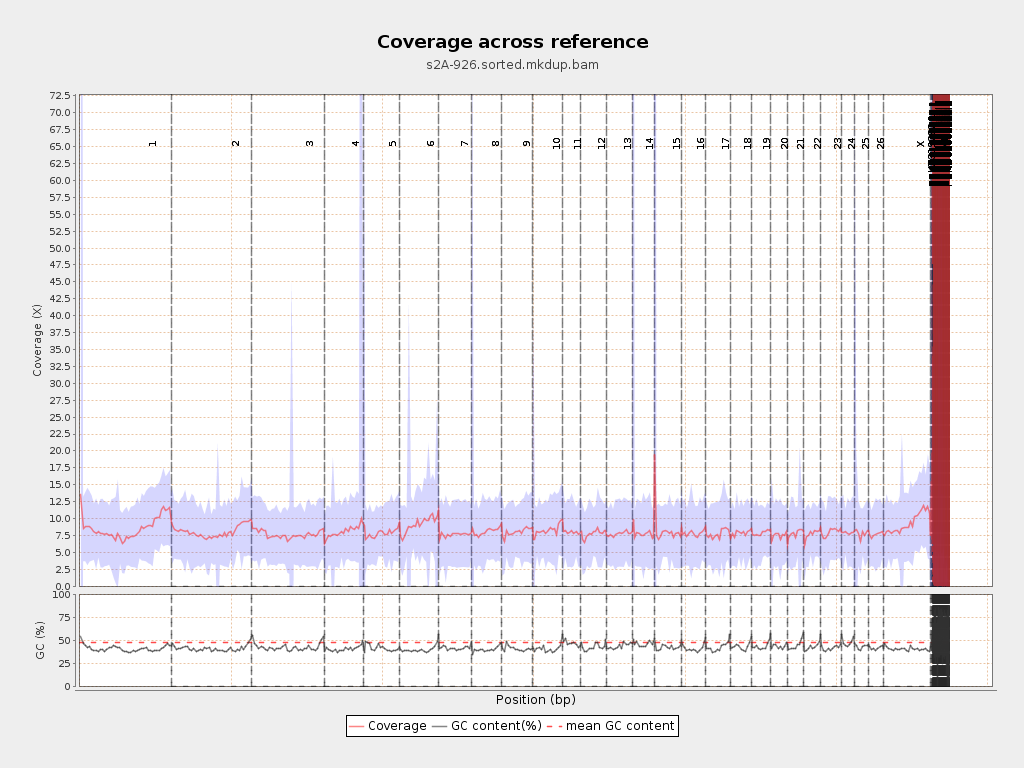

Supplement: Supplementary file 1 [file animals-14-00161-s001.zip › Supplementary Material 2/s2A-926/genome_coverage_across_reference.png]

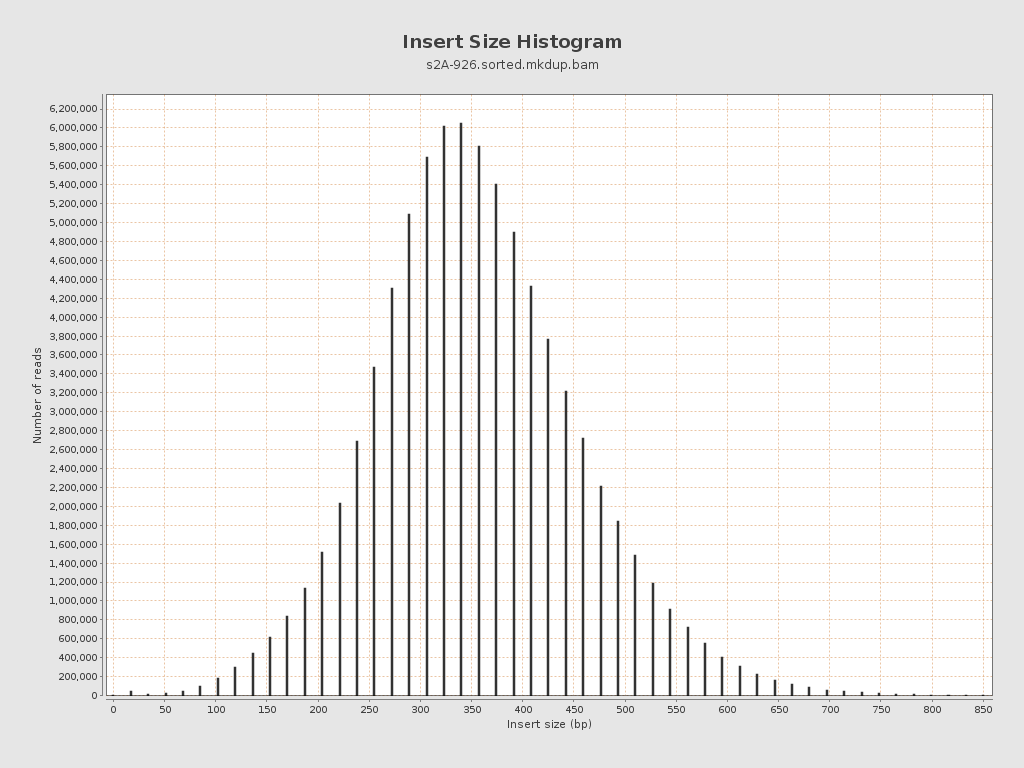

Supplement: Supplementary file 1 [file animals-14-00161-s001.zip › Supplementary Material 2/s2A-926/genome_insert_size_histogram.png]

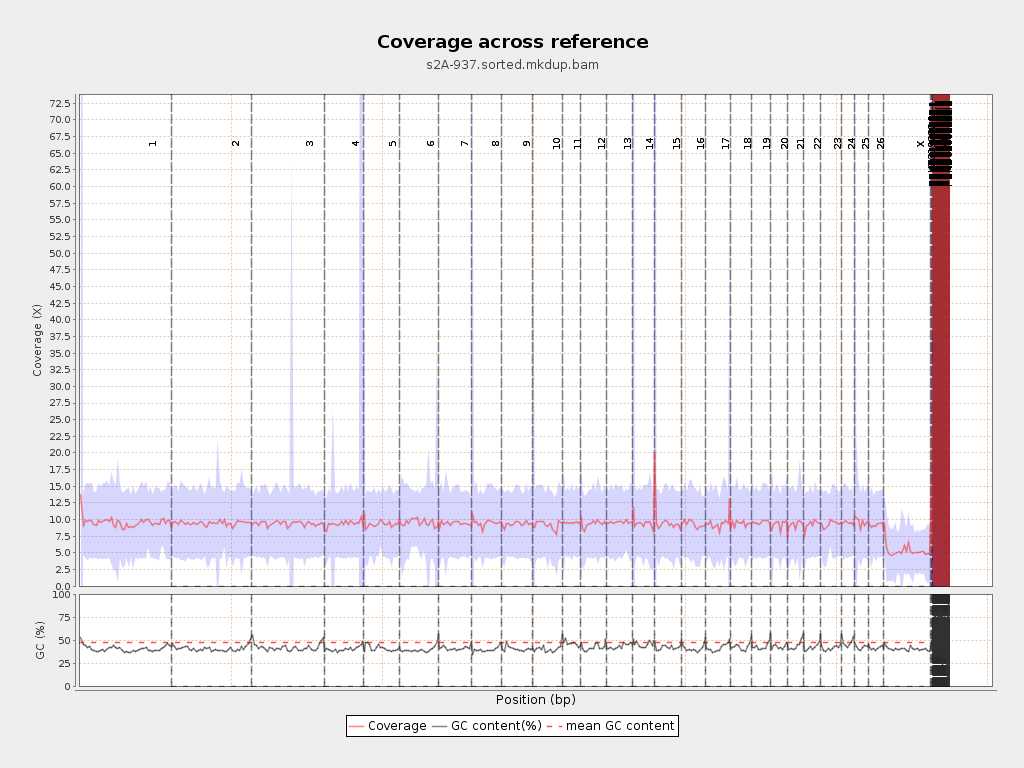

Supplement: Supplementary file 1 [file animals-14-00161-s001.zip › Supplementary Material 2/s2A-937/genome_coverage_across_reference.png]

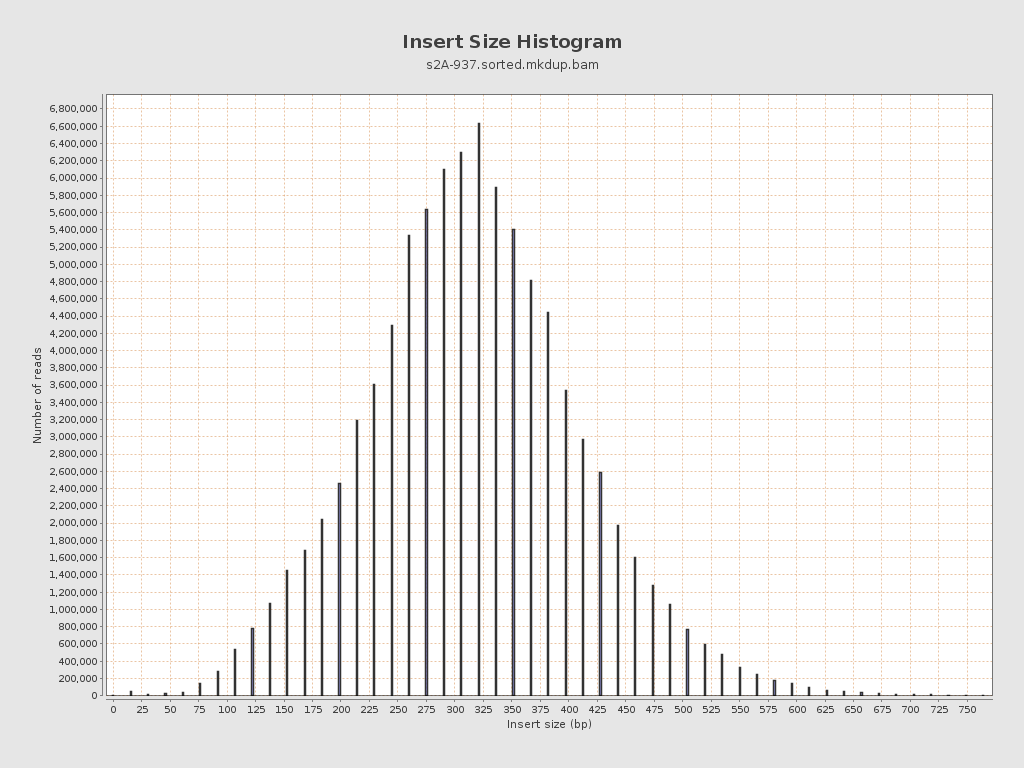

Supplement: Supplementary file 1 [file animals-14-00161-s001.zip › Supplementary Material 2/s2A-937/genome_insert_size_histogram.png]

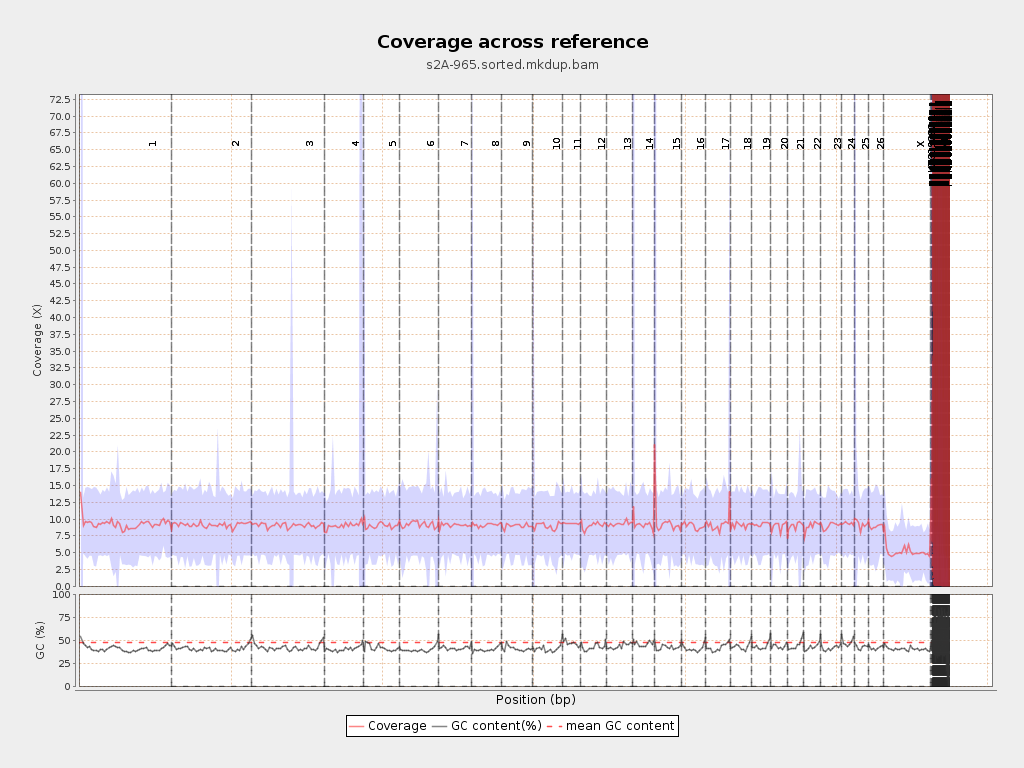

Supplement: Supplementary file 1 [file animals-14-00161-s001.zip › Supplementary Material 2/s2A-965/genome_coverage_across_reference.png]

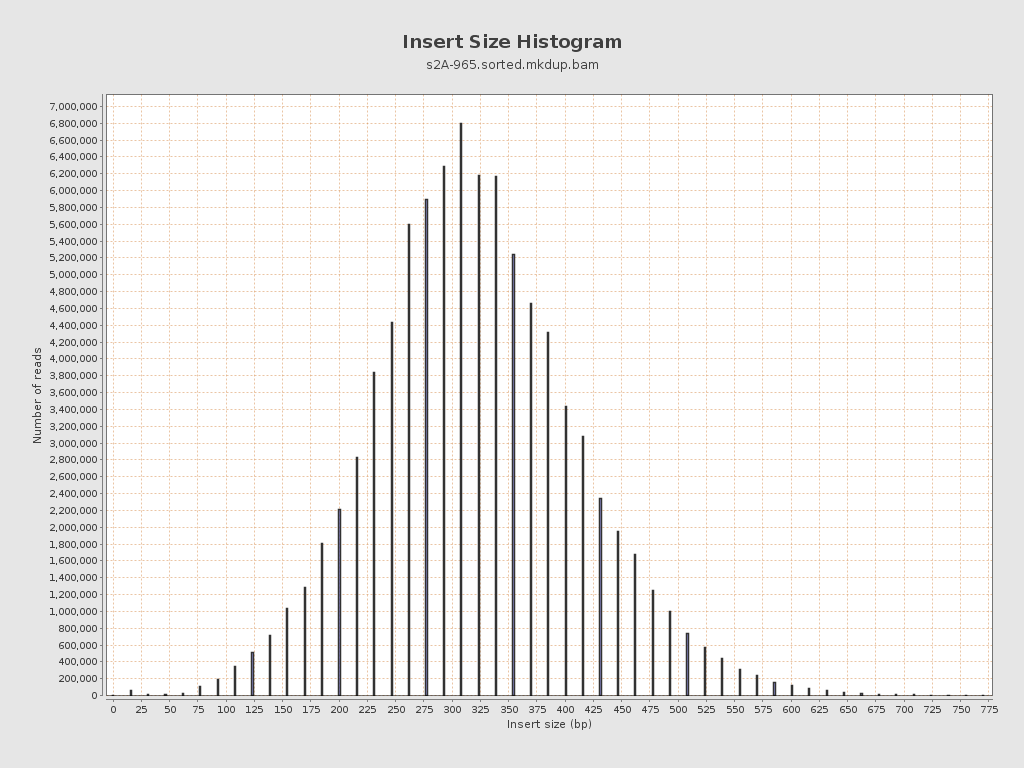

Supplement: Supplementary file 1 [file animals-14-00161-s001.zip › Supplementary Material 2/s2A-965/genome_insert_size_histogram.png]

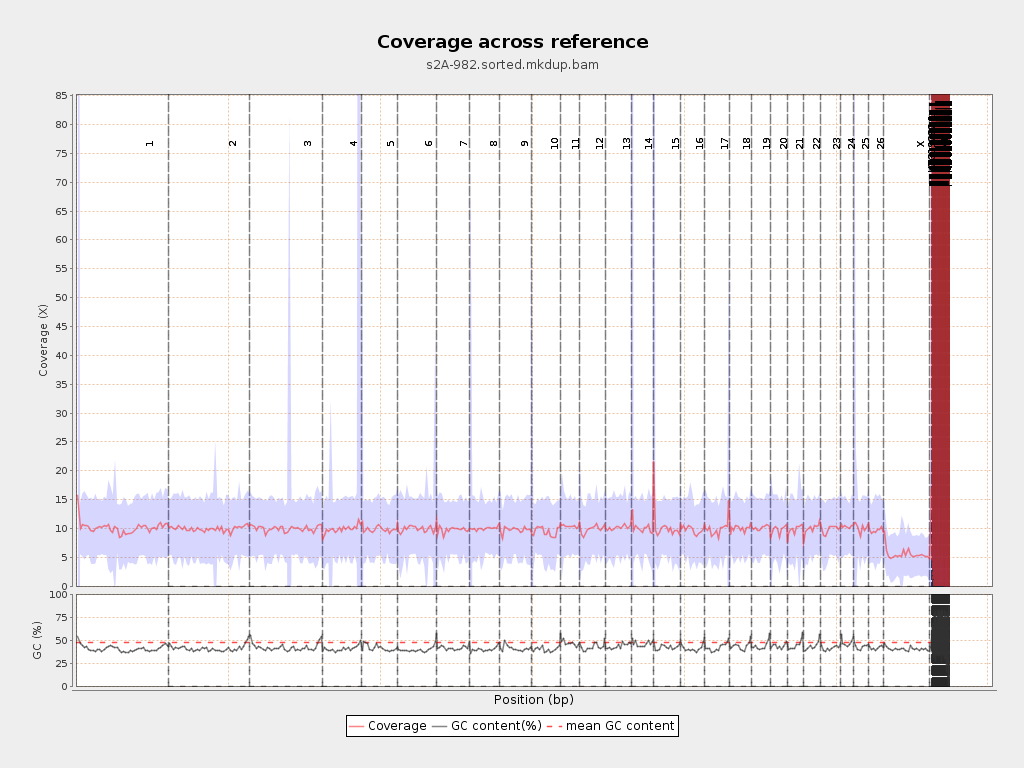

Supplement: Supplementary file 1 [file animals-14-00161-s001.zip › Supplementary Material 2/s2A-982/genome_coverage_across_reference.png]

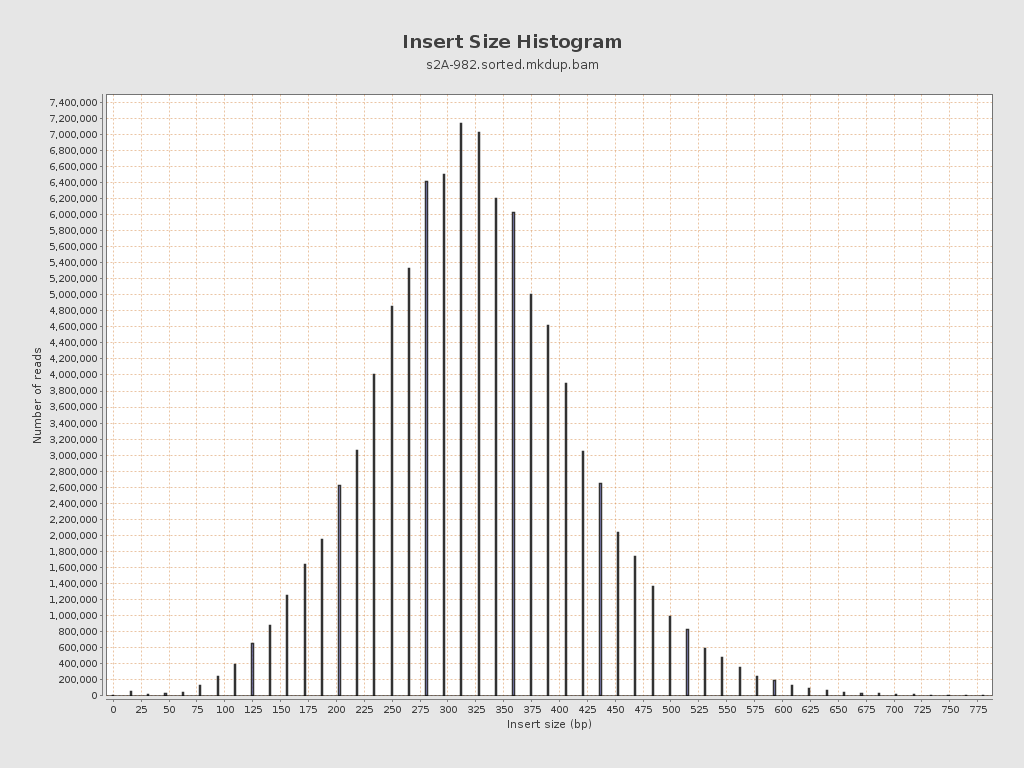

Supplement: Supplementary file 1 [file animals-14-00161-s001.zip › Supplementary Material 2/s2A-982/genome_insert_size_histogram.png]

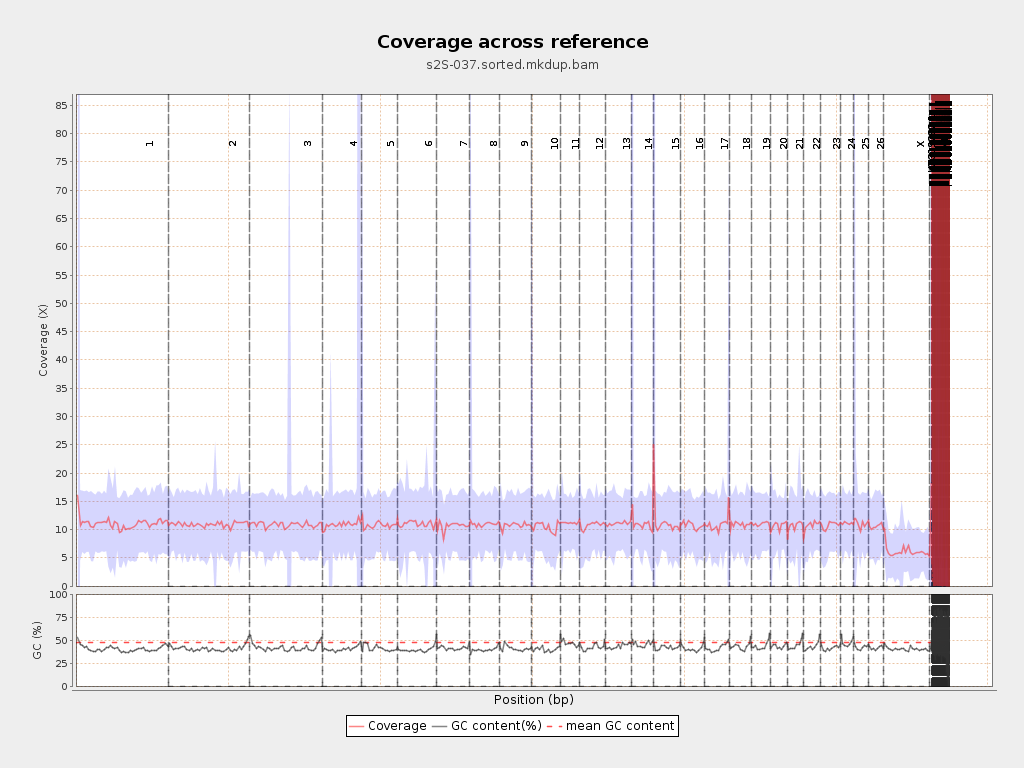

Supplement: Supplementary file 1 [file animals-14-00161-s001.zip › Supplementary Material 2/s2S-037/genome_coverage_across_reference.png]

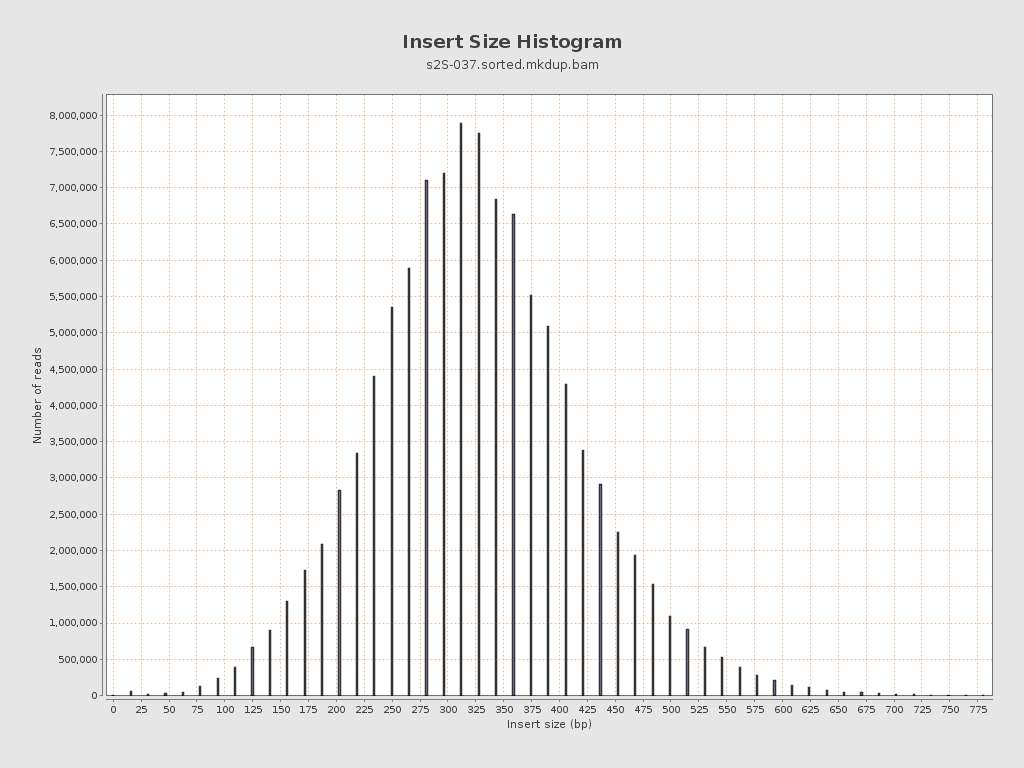

Supplement: Supplementary file 1 [file animals-14-00161-s001.zip › Supplementary Material 2/s2S-037/genome_insert_size_histogram.png]

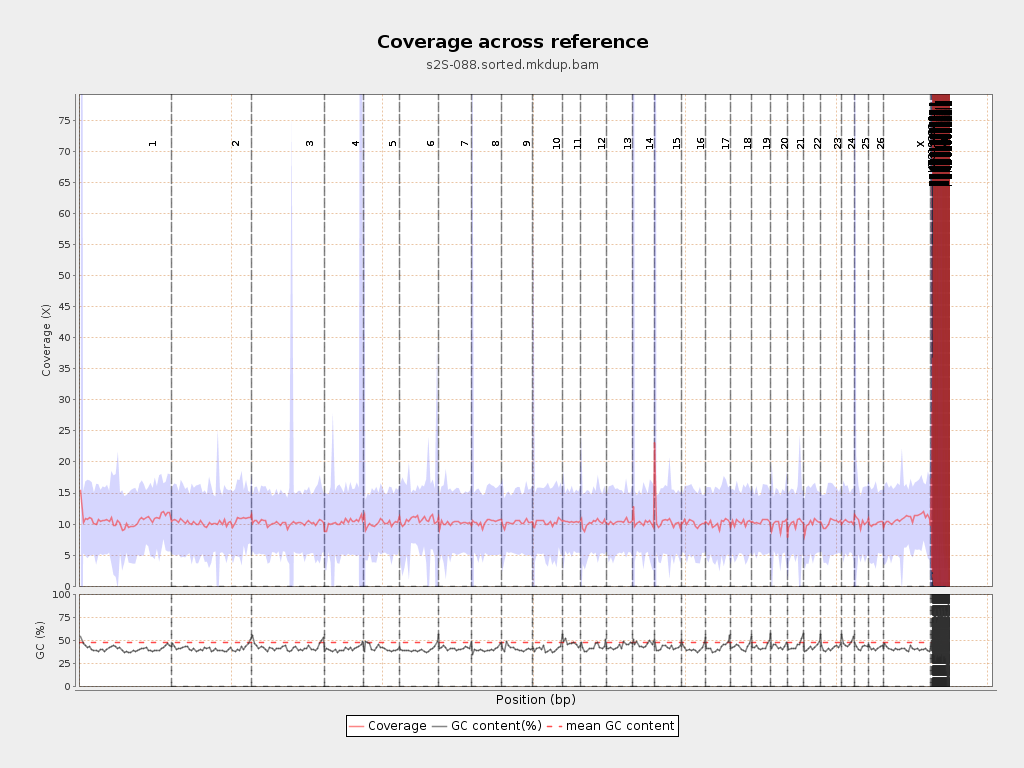

Supplement: Supplementary file 1 [file animals-14-00161-s001.zip › Supplementary Material 2/s2S-088/genome_coverage_across_reference.png]

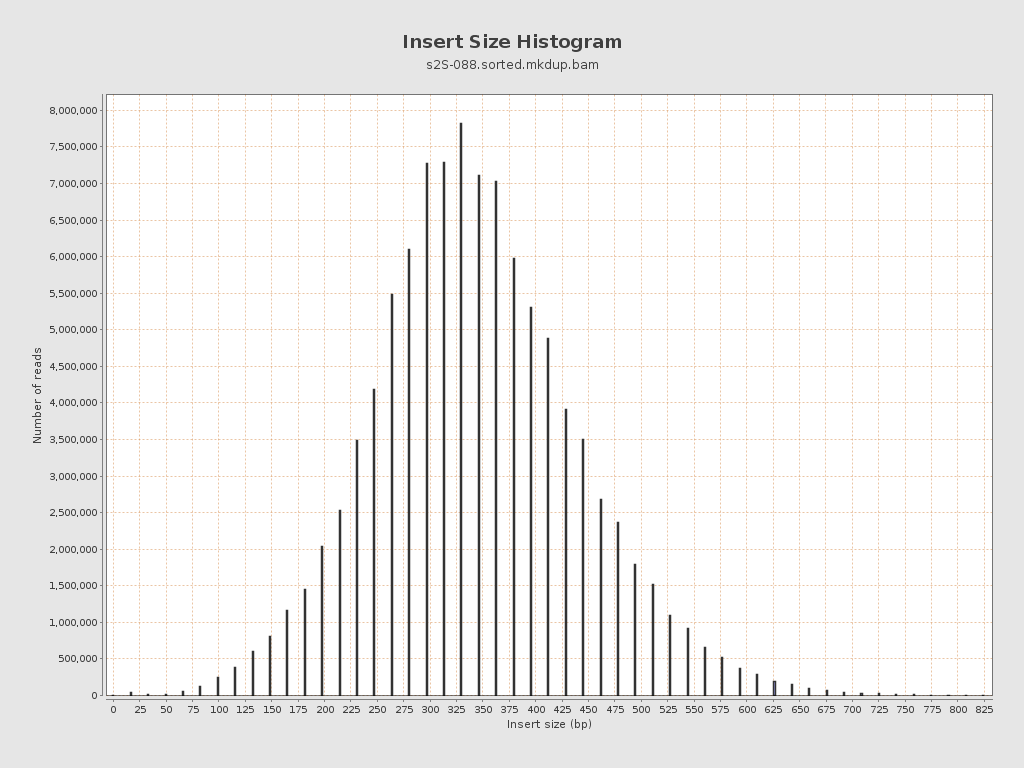

Supplement: Supplementary file 1 [file animals-14-00161-s001.zip › Supplementary Material 2/s2S-088/genome_insert_size_histogram.png]

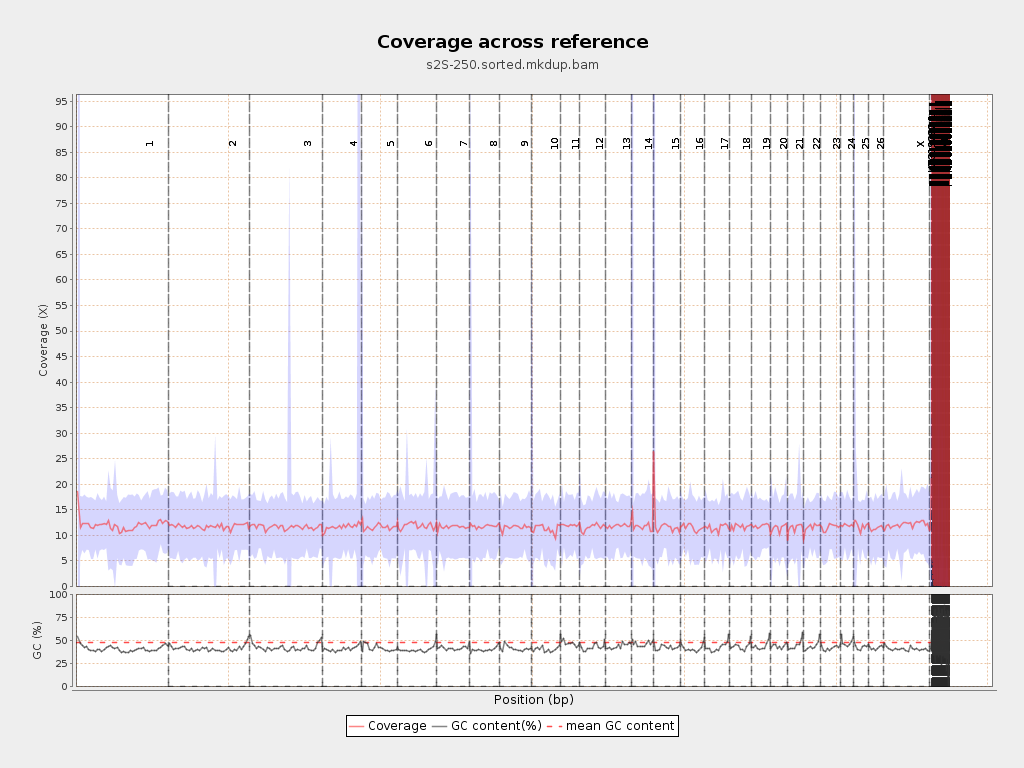

Supplement: Supplementary file 1 [file animals-14-00161-s001.zip › Supplementary Material 2/s2S-250/genome_coverage_across_reference.png]

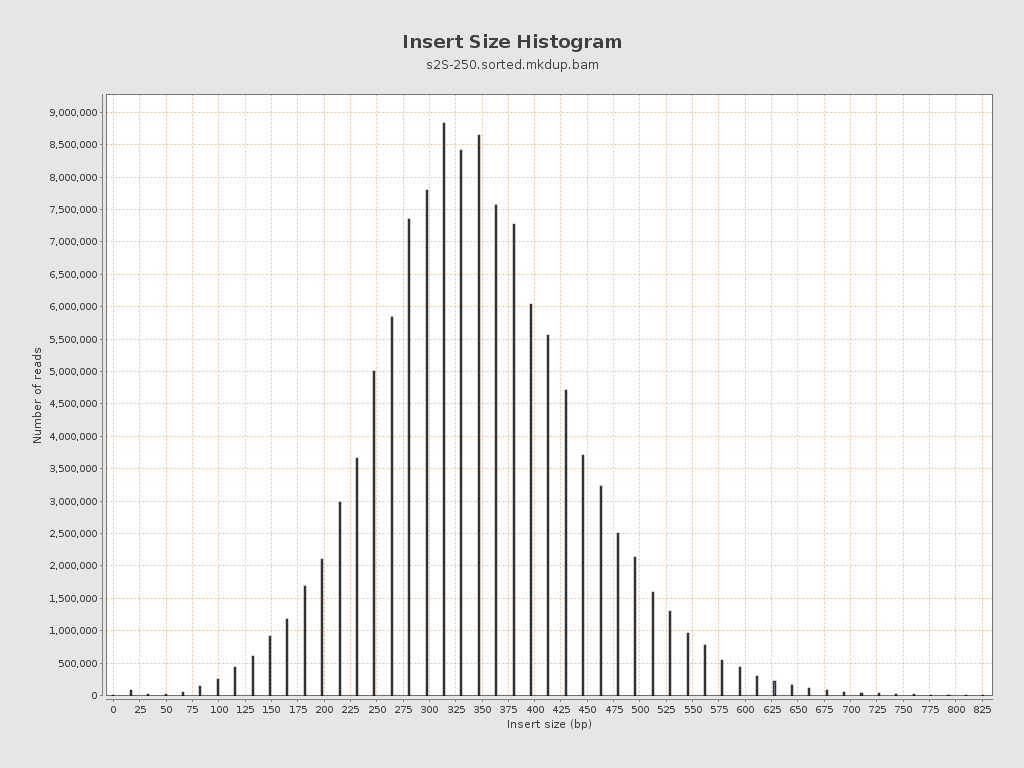

Supplement: Supplementary file 1 [file animals-14-00161-s001.zip › Supplementary Material 2/s2S-250/genome_insert_size_histogram.png]

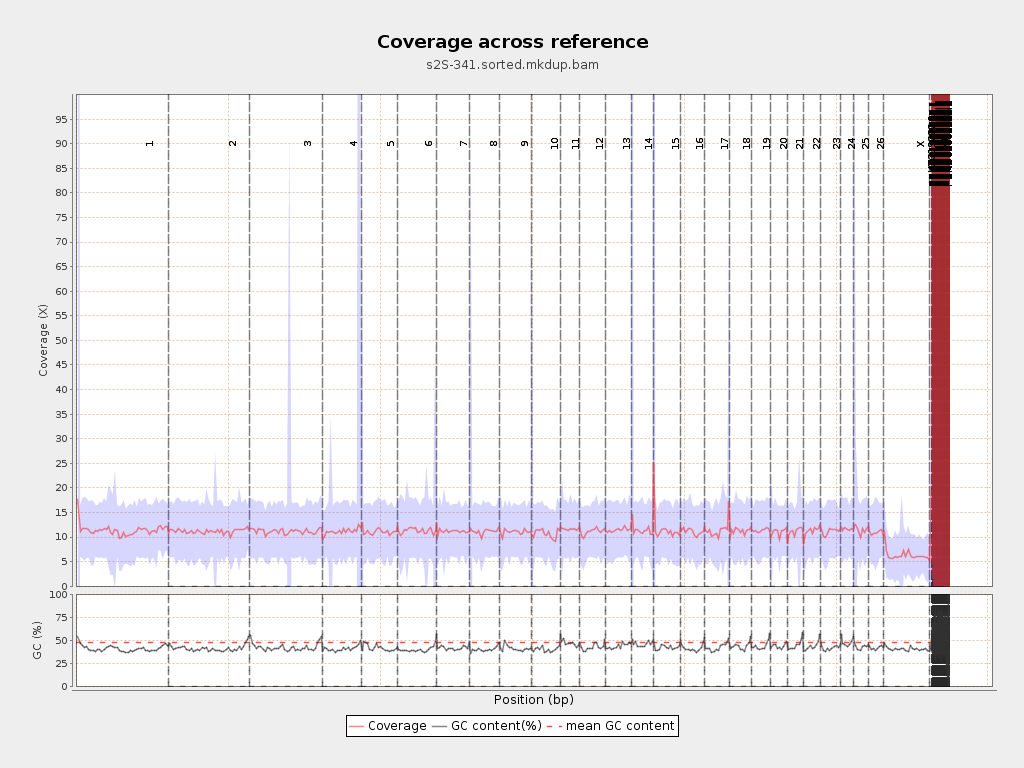

Supplement: Supplementary file 1 [file animals-14-00161-s001.zip › Supplementary Material 2/s2S-341/genome_coverage_across_reference.png]

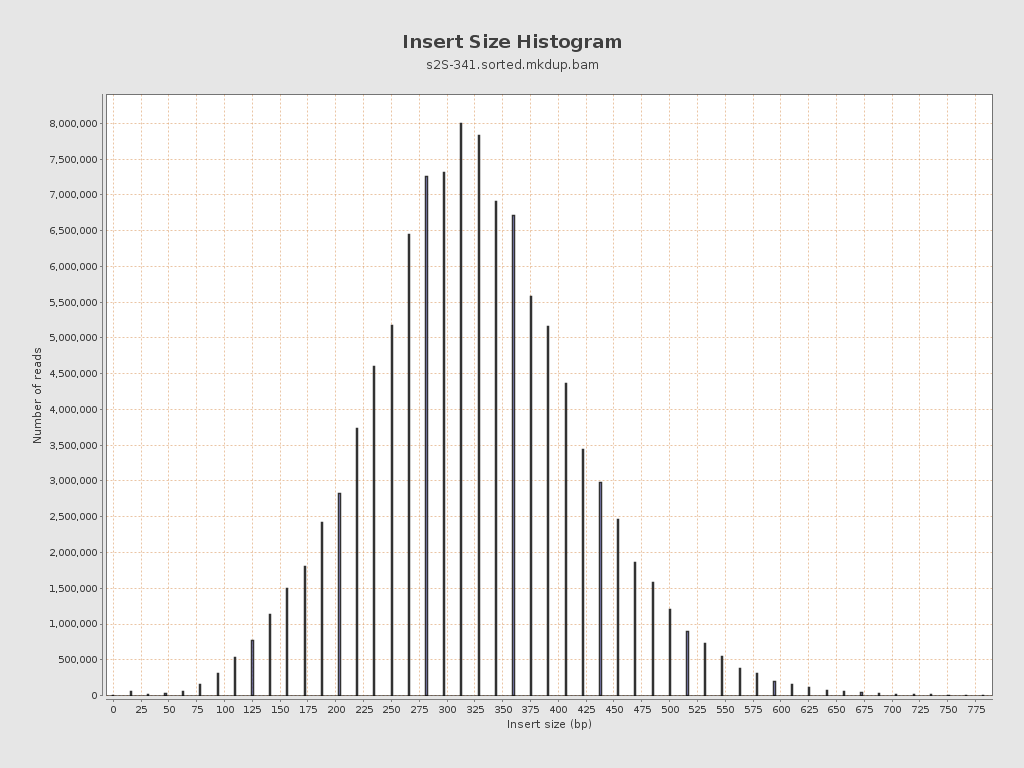

Supplement: Supplementary file 1 [file animals-14-00161-s001.zip › Supplementary Material 2/s2S-341/genome_insert_size_histogram.png]

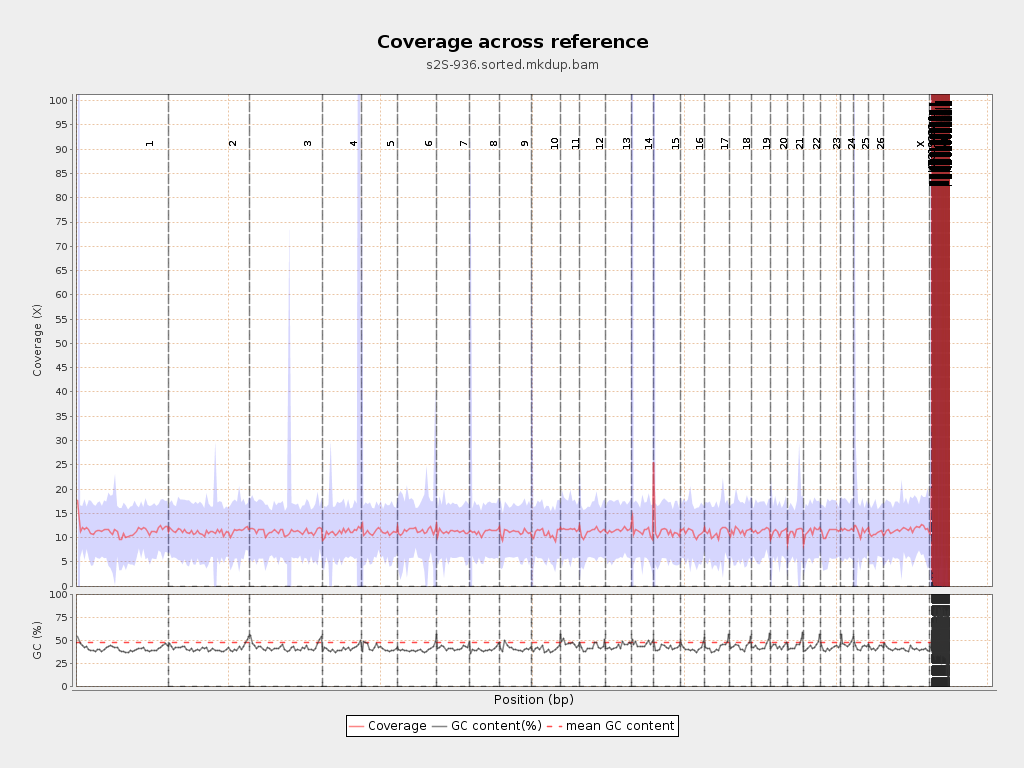

Supplement: Supplementary file 1 [file animals-14-00161-s001.zip › Supplementary Material 2/s2S-936/genome_coverage_across_reference.png]

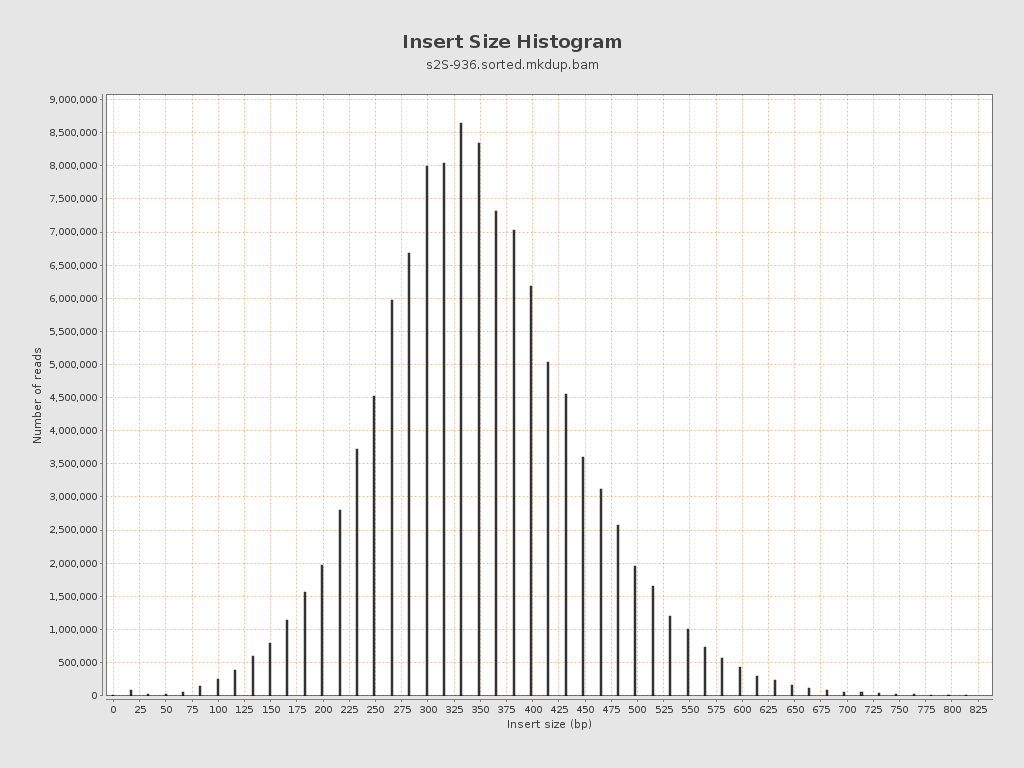

Supplement: Supplementary file 1 [file animals-14-00161-s001.zip › Supplementary Material 2/s2S-936/genome_insert_size_histogram.png]

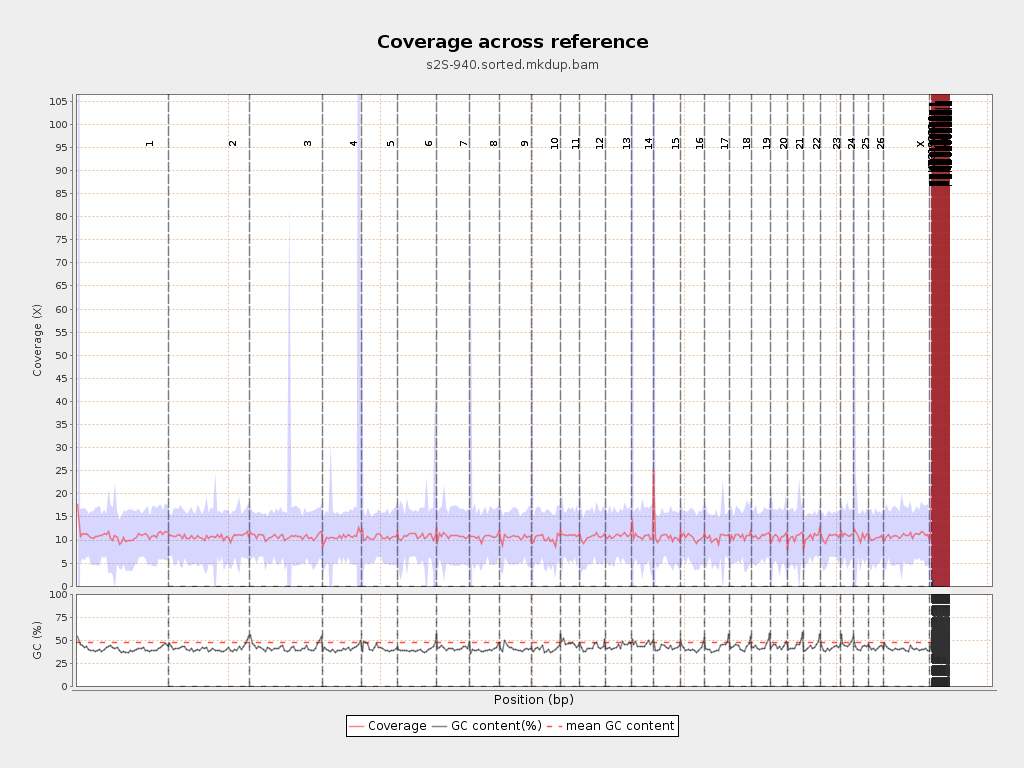

Supplement: Supplementary file 1 [file animals-14-00161-s001.zip › Supplementary Material 2/s2S-940/genome_coverage_across_reference.png]

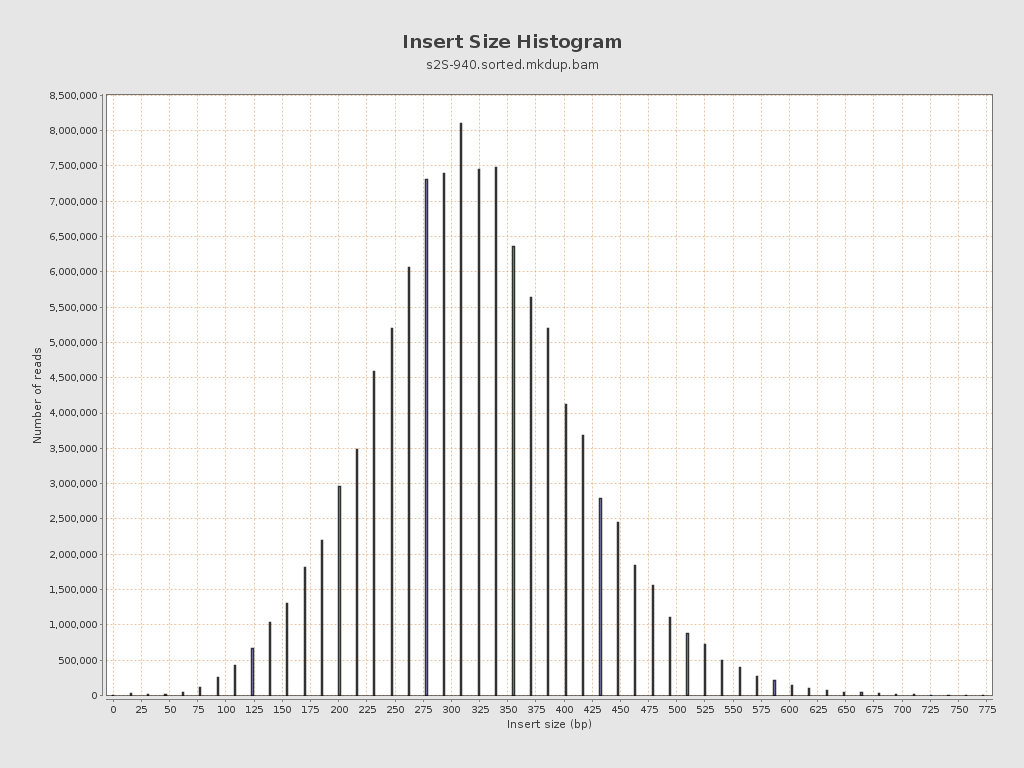

Supplement: Supplementary file 1 [file animals-14-00161-s001.zip › Supplementary Material 2/s2S-940/genome_insert_size_histogram.png]
